# Supplementary material for: Systematic review of ecological momentary assessment (EMA) studies of five public health-related behaviours: review protocol
Source: BMJ Open. 2021 Jul 16;11(7):e046435. doi: 10.1136/bmjopen-2020-046435 (PMC8287614; doi:10.1136/bmjopen-2020-046435)
Supplement: Supplementary data [file bmjopen-2020-046435supp002.pdf]

Supplementary Material 2 - Electronic search strategy

Ovid MEDLINE, Embase, PsycINFO

1. (ecological adj1 momentary adj1 assessment*).ti,ab.
2. (intensive adj1 longitudinal).ti,ab.
3. (ambulatory adj1 assessment*).ti,ab.
4. (experience adj1 sampl*).ti,ab.
5. (daily adj1 diar*).ti,ab.
6. (ecological adj1 momentary adj1 intervention).ti,ab.
7. within-person.ti,ab.
8. within-subject*.ti,ab.
9. (single adj1 case).ti,ab.
10. idiographic.ti,ab.
11. intraindividual.ti,ab.
12. 1 OR 2 OR 3 OR 4 OR 5 OR 6 OR 7 OR 8 OR 9 OR 10 OR 11
13. tobacco.ti,ab.
14. smok*.ti,ab.
15. cigarette.ti,ab.
16. alcohol*.ti,ab.
17. drinking.ti,ab.
18. addict*.ti,ab.
19. diet.ti,ab.
20. weight.ti,ab.
21. overweight.ti,ab.
22. obes*.ti,ab.
23. (healthy adj3 eat*).ti,ab.
24. physical activity.ti,ab.
25. exercise.ti,ab.
26. sedentary.ti,ab.
27. sitting.ti,ab.
28. leisure.ti,ab.
29. (sexual adj1 health).ti,ab.
30. condom.ti,ab.
31. contraceptive.ti,ab.
32. 13 OR 14 OR 15 OR 16 OR 17 OR 18 OR 19 OR 20 OR 21 OR 22 OR 23 OR 24 OR 25 OR 26 OR 27 OR 28 OR 29 OR 30 OR 31
33. 12 AND 32

Results: 18,014

Web of Science

1. TS=(ecological NEAR/1 momentary NEAR/1 assessment*)
2. TS=(ecological NEAR/1 momentary NEAR/1 intervention)
3. TS=(intensive NEAR/1 longitudinal)
4. TS=(ambulatory NEAR/1 assessment*)
5. TS=(experience NEAR/1 sampl*)
6. TS=(daily NEAR/1 diar*)
7. TS=(within-person or within-subject* or idiographic or intraindividual)

8. TS=(single NEAR/1 case)
9. 1 OR 2 OR 3 OR 4 OR 5 OR 6 OR 7 OR 8
10. TS=(healthy NEAR/1 eat*)
11. TS=(sexual NEAR/1 health)
12. TS=(smok* or tobacco* or cigarette or alcohol* or drinking or addict* or diet or weight or overweight or obes* or physical activity or exercise or sedentary or leisure or sitting or condom or contraceptive)
13. 10 OR 11 OR 12
14. 9 AND 13

Results: 11,036
