# Supplementary material for: Systematic review of ecological momentary assessment (EMA) studies of five public health-related behaviours: review protocol
Source: BMJ Open. 2021 Jul 16;11(7):e046435. doi: 10.1136/bmjopen-2020-046435 (PMC8287614; doi:10.1136/bmjopen-2020-046435)
Supplement: Author's manuscript [file bmjopen-2020-046435.draft_revisions.pdf]

BMJ Open is committed to open peer review. As part of this commitment we make the peer review history of every article we publish publicly available.

When an article is published we post the peer reviewers' comments and the authors' responses online. We also post the versions of the paper that were used during peer review. These are the versions that the peer review comments apply to.

The versions of the paper that follow are the versions that were submitted during the peer review process. They are not the versions of record or the final published versions. They should not be cited or distributed as the published version of this manuscript.

BMJ Open is an open access journal and the full, final, typeset and author-corrected version of record of the manuscript is available on our site with no access controls, subscription charges or pay-per-view fees (http://bmjopen.bmj.com).

If you have any questions on BMJ Open's open peer review process please email info.bmjopen@bmj.com

BMJ Open

Systematic review of Ecological Momentary Assessment (EMA) studies of five public health-related behaviours: Review Protocol.

Journal:	*BMJ Open*
Manuscript ID	bmjopen-2020-046435
Article Type:	Protocol
Date Submitted by the Author:	02-Nov-2020
Complete List of Authors:	Kwasnicka, Dominika; SWPS University of Social Sciences and Humanities Wroclaw Campus Library, Faculty of Psychology<br>Kale, Dimitra; University College London<br>Schneider, Verena; University College London<br>Keller, Jan; Freie Universität Berlin<br>Yeboah-Asiamah Asare, Bernard; Curtin University<br>Powell, Daniel; University of Aberdeen<br>Naughton, Felix; University of East Anglia Faculty of Medicine and Health Sciences, School of Health Sciences; University of East Anglia<br>ten Hoor, Gill A. ; Department of Work & Social Psychology, Maastricht University,<br>Verboon, Peter; Open Universiteit Nederland Faculteit Managementwetenschappen<br>Perski, Olga; University College London, Department of Behavioural Science and Health
Keywords:	PUBLIC HEALTH, STATISTICS & RESEARCH METHODS, SOCIAL MEDICINE, SPORTS MEDICINE, NUTRITION & DIETETICS

SCHOLARONE™
Manuscripts

I, the Submitting Author has the right to grant and does grant on behalf of all authors of the Work (as defined in the below author licence), an exclusive licence and/or a non-exclusive licence for contributions from authors who are: i) UK Crown employees; ii) where BMJ has agreed a CC-BY licence shall apply, and/or iii) in accordance with the terms applicable for US Federal Government officers or employees acting as part of their official duties; on a worldwide, perpetual, irrevocable, royalty-free basis to BMJ Publishing Group Ltd ("BMJ") its licensees and where the relevant Journal is co-owned by BMJ to the co-owners of the Journal, to publish the Work in this journal and any other BMJ products and to exploit all rights, as set out in our licence.

The Submitting Author accepts and understands that any supply made under these terms is made by BMJ to the Submitting Author unless you are acting as an employee on behalf of your employer or a postgraduate student of an affiliated institution which is paying any applicable article publishing charge ("APC") for Open Access articles. Where the Submitting Author wishes to make the Work available on an Open Access basis (and intends to pay the relevant APC), the terms of reuse of such Open Access shall be governed by a Creative Commons licence – details of these licences and which Creative Commons licence will apply to this Work are set out in our licence referred to above.

Other than as permitted in any relevant BMJ Author's Self Archiving Policies, I confirm this Work has not been accepted for publication elsewhere, is not being considered for publication elsewhere and does not duplicate material already published. I confirm all authors consent to publication of this Work and authorise the granting of this licence.

For peer review only

Title: Systematic review of Ecological Momentary Assessment (EMA) studies of five public health-related behaviours: Review Protocol.

Authors: Dominika Kwasnicka[1,2], Dimitra Kale[3], Verena Schneider[3], Jan Keller[4], Bernard Yeboah-Asiamah Asare[5,6], Daniel Powell[6,7], Felix Naughton[8], Gill ten Hoor[9], Peter Verboon[10], Olga Perski[3]

1. Faculty of Psychology, SWPS University of Social Sciences and Humanities, Aleksandra Ostrowskiego 30b, 53-238, Wroclaw, Poland
2. NHMRC CRE in Digital Technology to Transform Chronic Disease Outcomes, Melbourne School of Population and Global Health, University of Melbourne, 333 Exhibition Street, 3000, Melbourne, Australia
3. Department of Behavioural Science and Health, Institute of Epidemiology and Health, Faculty of Population Health Sciences, University College London, United Kingdom
4. Department of Education and Psychology, Freie Universität Berlin, Berlin, Germany
5. School of Public Health, Curtin University, Kent Street, 6102, Perth, Australia
6. Health Psychology, Institute of Applied Health Sciences, University of Aberdeen, Aberdeen, AB25 2ZD, United Kingdom
7. Rowett Institute, University of Aberdeen, Aberdeen, AB25 2ZD, United Kingdom
8. Behavioural and Implementation Science Research Group, School of Health Sciences, University of East Anglia, Norwich, NR4 7UL, United Kingdom
9. Department of Work and Social Psychology, Faculty of Psychology and Neurosciences, Maastricht University, Maastricht, The Netherlands

For peer review only - http://bmjopen.bmj.com/site/about/guidelines.xhtml

10. Faculty of Psychology, Open University, Heerlen, The Netherlands

Corresponding author: Dominika Kwasnicka, Faculty of psychology SWPS University of Social Sciences and Humanities, Aleksandra Ostrowskiego 30b, 53-238, Wrocław and NHMRC CRE in Digital Technology to Transform Chronic Disease Outcomes, Melbourne School of Population and Global Health, University of Melbourne, 333 Exhibition Street, 3000, Melbourne, Australia. E-mail: dkwasnicka@swps.edu.pl

Word count: 2,958

Keywords: ambulatory assessment, Ecological Momentary Assessment, EMA, experience sampling, health, psychology, systematic review, within-person design

For peer review only

ABSTRACT

Introduction: Ecological Momentary Assessment (EMA) involves repeated, real-time assessments of phenomena (e.g., cognitions, emotions, behaviours) over a period of time in naturalistic settings. EMA is increasingly used to study both within- and between-person processes. We will review EMA studies investigating key health behaviours and synthesise: 1) study characteristics (e.g., frequency of assessments, adherence, incentives), 2) associations between psychological predictors and behaviours or behaviour-related outcomes, and 3) moderators of adherence to EMA protocols.

Methods and analysis: This review will focus on EMA studies conducted across five public health behaviours in adult, non-clinical populations: physical activity, dietary behaviour, alcohol consumption, tobacco smoking, and preventive sexual health behaviours. Studies need to have assessed at least one psychological or contextual predictor of these behaviours. Studies reporting exclusively on physiological outcomes (e.g., cortisol) or not conducted under free-living conditions will be excluded. We will search OVID Medline, Embase, PsycINFO and Web of Science using terms relevant to EMA and the selected health behaviours. Reference lists of existing systematic reviews of EMA studies will be hand searched. Identified articles will be screened by two reviewers. This review is expected to provide a comprehensive summary of EMA studies assessing psychological predictors of five public health behaviours.

Ethics and dissemination: The results will be disseminated through peer-reviewed publications and presentations. Data from included studies will be made available to other researchers. No ethics are required.

Review registration: The review protocol has been registered with PROSPERO 2020 CRD42020168314. Available from: www.crd.york.ac.uk/prospero/display_record.php?ID=CRD42020168314.

For peer review only - http://bmjopen.bmj.com/site/about/guidelines.xhtml

Strengths and limitations of this study:

- This systematic review will identify and synthesise evidence from EMA studies across five key public health behaviours in adult, non-clinical populations, including physical activity, healthy eating, alcohol consumption, tobacco smoking, and preventive sexual health behaviours.
- We will review characteristics of EMA studies (e.g., length of follow-up, assessment type, intensity, adherence rates) and associations between psychological predictors and behaviours or behaviour-related outcomes, examining rates of adherence to EMA protocols across different health behaviours and moderators of adherence (e.g., study setting, type of behaviour).
- This review is expected to inform design decisions in future observational EMA studies and just-in-time adaptive interventions aimed at understanding and improving health behaviours.
- Our comprehensive search strategy is likely to result in a large number of included studies; extracted data will be made available to other researchers, thus allowing for the exploration of additional research questions and potential for setting up a 'living review'.
- As included studies are likely to be heterogeneous, this may limit the overarching conclusions that can be drawn, and will likely prevent meta-analysis combining effect sizes from multiple studies and across all behaviours.

For peer review only

INTRODUCTION

Ecological Momentary Assessment (EMA), also known as ambulatory assessment or experience sampling methodology, involves repeated, real-time assessments of phenomena (e.g., cognitions, emotions and behaviours) over a period of time in naturalistic settings (1). EMA is increasingly used to study within- and between-person processes, including associations between psychological and health behaviour-related variables (e.g., positive affect and physical activity in general population or stress and lapse risk in smokers attempting to stop). For researchers and healthcare professionals to understand and change behaviour, it is important for theories and interventions to be applicable to both momentary states of individuals (within-person processes) and groups of individuals (between-person processes) (2). Despite the popularity and importance of EMA for studying health-related behaviours, there has been no comprehensive systematic investigation of characteristics of EMA studies (e.g., rates of adherence, length of follow-ups, incentive schedules) and potential moderators of adherence (e.g., study setting, type of health behaviour), with attempts to describe associations between psychological predictors and behaviours or behaviour-related outcomes across key public health behaviours.

Previous reviews of EMA studies have focused on clinical conditions such as borderline personality disorder (3), psychotic disorder (4), mood disorders (5), binge eating (6), bulimia nervosa (7), anxiety disorder (8), schizophrenia (9), alcohol use disorder (10), chronic pain (11), and specific populations such as children and adolescents (12), youth (13) and older adults (14). Health behaviour-specific reviews of EMA studies have focused on physical activity (15,16), sedentary behaviour (16), alcohol use (17), craving and substance use (18), dietary behaviours (19), and the relationship between alcohol use and sexual decision making (20). Previous EMA reviews have also focused on interrelations between specific psychological

For peer review only - http://bmjopen.bmj.com/site/about/guidelines.xhtml

variables, such as the association of everyday social interactions with intra-individual variability in affect (21).

While systematic reviews of EMA studies focusing on specific health behaviours have been conducted (18,22,23), there are no overarching reviews that can help address broad questions about study characteristics (e.g., length of follow-up, frequency of EMAs, adherence, incentive schedules), rates and moderators of adherence (e.g., study setting, participant characteristics) and predictor-behaviour and predictor-outcome associations across different health behaviours and potential moderators (e.g., study setting, study quality). It is expected that this review will help fill this gap. We also expect that this review will help inform the design of future EMA studies by providing a summary of best practice across research contexts, settings and health-related behaviours. For instance, results may be useful for informing researchers' understanding of what frequency or intensity of change we would expect to see at what temporal resolution [i.e., informed by a 'theory of change' (24)], which can then inform assessment scheduling decisions. This review is likely to include a large number of studies, thus providing a comprehensive overview of the EMA literature.

The current study

We will synthesise evidence from EMA studies that report either within- or between-person predictor-behaviour or predictor-outcome associations. The review will focus on five key public health behaviours: (1) physical activity (including sedentary behaviour), (2) healthy eating, (3) alcohol consumption, (4) tobacco smoking and (5) sexual health behaviours (including contraceptive use).

The review aims are:

1. To summarise adherence to EMAs, total length of data collection of EMAs, prompting frequency of EMAs, and incentives structures across studies.

For peer review only

2. To describe within- and between-person predictor-behaviour and predictor-outcome associations across EMA studies (e.g., associations between intention and behaviour).
3. To assess potential moderators of adherence to EMAs (e.g., study setting, participant characteristics).

METHODS AND ANALYSIS

Study design

This review will adhere to the Preferred Reporting Items for Systematic Reviews and Meta-Analyses (PRISMA) checklist (Supplementary Material 1).

Inclusion criteria

This review will focus on five key public health behaviours in healthy adults (i.e. non-clinical populations) aged 18+ years, namely:

1. physical activity, including studies addressing sedentary behaviour;
2. dietary behaviour, including snacking or fruit and vegetable consumption
3. alcohol consumption;
4. tobacco smoking, including cigarettes, cigars or pipe;
5. sexual health behaviours, including contraceptive/condom use.

No restrictions on geographical location or publication date will be set. To be included, studies need to incorporate multiple (i.e., two or more) within-day, daily or weekly assessments of predictors, behaviours or behavioural outcomes (e.g., weight loss) and to have reported either (or both) within- or between-person predictor-behaviour or predictor-outcome associations. To be included, studies needed to assess one of the aforementioned behaviours and at least one psychological or contextual variable via EMAs.

In addition to self-report measures, included studies can use objective or physiological measures for psychological predictors (e.g., cortisol or heart rate variability to measure stress) and behavioural outcomes (e.g., accelerometer data to measure physical activity). Studies

For peer review only - http://bmjopen.bmj.com/site/about/guidelines.xhtml

reporting associations between behaviours and psychological consequences (e.g., whether physical activity predicts affect) will be included providing that they also report psychological or contextual predictor-behaviour associations (e.g., whether positive affect predicts physical activity). We will include individuals with overweight and obesity given that 39% of adults globally fall into this category, with most Western countries averaging above 50% (25). Studies including participants with a diagnosed mental or physical health condition who were not recruited into the study on the basis of their condition will be included (e.g., studies including participants with clinical levels of depression but where this was not an inclusion criterion). Studies in which a behavioural or pharmacological intervention was delivered will be included providing that participants were asked to complete free-living EMAs.

Exclusion criteria

Studies only reporting physiological outcomes (e.g., cortisol or heart rate variability to measure stress) will not be included. Laboratory studies will not be included. Studies including clinical populations and recruiting participants on the basis of being diagnosed with a physical or mental health condition such as cancer, cardiovascular disease, depression, binge eating disorder or substance use disorder (also including case-control studies) will be excluded. Studies only focusing on purchasing behaviours (e.g., tobacco purchasing, food purchasing) will not be included. Studies not published in English or where no full text could be obtained will also not be included. Although behaviour-behaviour associations may also be considered relevant, our electronic search is not designed to capture such studies, and behaviour-behaviour associations will hence not be considered further in this review.

Search methods for the identification of studies

Electronic searches

We will search Ovid MEDLINE, Embase, PsycINFO and Web of Science (see Supplementary Material 2 for the full search strategy). Terms will be searched in titles and abstracts as free

For peer review only

text terms or as index terms (e.g., Medical Subject Headings), as appropriate. We will combine two groups of terms, the first with terms relevant to EMAs and within-person study designs; the second with terms relevant to the five health behaviours addressed in this review.

Example terms used:

1. (ecological adj1 momentary adj1 assessment*) OR (intensive adj1 longitudinal) OR (ambulatory adj1 assessment*) OR (experience adj1 sampl*) OR (daily adj1 diar*) OR (ecological adj1 momentary adj1 intervention) OR within-person OR within-subject* OR (single adj1 case) OR idiographic
2. tobacco OR smok* OR alcohol* OR (healthy adj3 eat*) OR diet OR weight OR overweight OR obes* OR physical activity OR exercise OR (medication adj1 adherence) OR (treatment adj1 adherence) OR (sexual adj1 health) OR condom OR contraceptive
3. 1 AND 2

Electronic and hand searches were conducted in January 2020. We restricted the search to human studies available in English that are published in peer-reviewed journals (Online Attachment 2).

Searching for other sources

Reference lists of existing systematic reviews of EMA studies will be hand searched and expertise within the review team will be used to identify additional articles of interest.

Data collection and analysis

Selection of studies

Identified articles will be merged using Covidence (26) and duplicate records will be removed. The two lead authors (DK and OP) will independently screen titles and abstracts (yes, maybe, no) against the pre-specified inclusion criteria. Full texts will be screened by two reviewers independently (yes, no); discrepancies will be resolved by the lead authors and inclusion will

For peer review only - http://bmjopen.bmj.com/site/about/guidelines.xhtml

be further discussed with other team members if needed. In line with the PRISMA checklist, key reasons for exclusion will be recorded at the full text stage. These will include: lack of psychological predictors or outcomes; study not being relevant to the five key public health behaviours of interest; wrong study design (not an EMA study); participants being recruited based on a health condition (i.e., clinical population); participants younger than 18 years old; studies of purchasing behaviours; conference abstracts; protocols; duplicates; studies not published in English or no full text could be obtained. We will follow the hierarchy of the exclusion criteria, listing the first reason from the aforementioned list as the key reason for exclusion.

Data extraction and management

A data extraction form will be developed in Microsoft Excel by the two lead authors in collaboration with the study team to extract information and to import data into R for analysis. Each study will be allocated a unique study identification number. Data will be extracted on:

- *Study description* (study author, year, country, study funder);
- *Participant characteristics* (sample size, mean or median age (SD); gender (% female); educational attainment (% university education); population type (e.g., men who have sex with men, older adults), ethnicity (% White ethnicity);
- *Health behaviour(s) assessed* (e.g., physical activity, dietary behaviour, tobacco smoking); and how the health behaviour(s) were measured (e.g., daily/hourly step count, number of cigarettes smoked);
- *Behavioural outcomes* (e.g., weight loss) and how they were measured (e.g., weekly weigh-ins with Wi-Fi connected scales);
- *Psychological predictors* (e.g., intentions, self-efficacy) and how they were measured (e.g., method, measurement frequency, whether the measure was developed for the study, whether a single or multiple items were used);

For peer review only - http://bmjopen.bmj.com/site/about/guidelines.xhtml

- *EMA study type* (e.g., observational, interventional, both);
- *EMA delivery mode* (e.g., mobile phone, website/online, pen-and-paper);
- *EMA method* (e.g., signal contingent, event contingent);
- *EMA characteristics* (e.g., total study duration in days; prompting frequency (e.g., hourly, daily, weekly), incentive schedule (e.g., flat rate, payment per EMA);
- *Adherence to EMA* (e.g., % assessments completed out of available prompts);

For each study, one reviewer will extract the data. Twenty percent of studies will be double checked for accuracy and completeness by a second reviewer.

Quality appraisal

Included studies may vary in quality, which will be considered through a quality appraisal. The appraisal tool was developed by the review team, based on an existing EMA reporting checklist (27), and includes the following five criteria: 1) rationale for EMA design, 2) prior power analysis to determine sample size, 3) percentage adherence to the EMA protocol, 4) treatment of missingness, and 5) level of data aggregation. Moreover, we will apply a standardized classification system based on the Effective Public Health Practice Project quality assessment tool (28,29) by rating the quality of each EMA study as strong (≥3 strong ratings and no weak ratings), moderate, or weak (≥3 weak ratings and no strong rating) (Table 1). The five quality indicators will be coded by one reviewer, with 20% double checked by a second reviewer. Discrepancies will be resolved through discussion among the lead authors. Where possible, study quality will be entered as a moderator of predictor-behaviour or predictor-outcome associations.

Topic: **Factors**	**Strong**	**Moderate**	**Weak**
	**Rationale**		
***Rationale for EMA design provided**:* Why was an EMA design chosen to examine the research question?	A strong rationale provided for the EMA design of predictor AND behaviour/ outcome	Rationale provided but not very strong for the EMA design of either the	No rationale for the EMA design regarding predictor and

		predictor OR behaviour/ outcome	behaviour/ outcome
**Power analysis, sample size and participant adherence**			
***Power analysis**:* A priori power analysis to determine sample size	An a priori power analysis is reported and the enrolled sample size met power analysis indication / OR: sufficient explanation as to why an a priori power analysis was not needed	An a priori power analysis is reported but sufficient sample size/number of observations was not achieved	No information about power analysis / OR: a post-hoc power analysis is reported
Adherence to EMA protocol: Percentage of answered EMA prompts across all participants for the main EMA study period	Percentage of answered EMA prompts >80%	Percentage of answered EMA prompts 60-79.99%	Percentage of answered EMA prompts less than 60%
**Data analysis**			
Treatment of missingness: Report whether study dropout or non-adherence to EMAs (e.g., missed prompts) are related to specific variables	Missing mechanisms/predictors are identified, reported and mitigated for if needed	Missing mechanisms/predictors are identified and reported but not mitigated for	Missing mechanisms/predictors are not identified or reported
Level of aggregation in data analysis: Data underpinning the predictor and behaviour/outcome are aggregated (vs. maintained at the within-person level)	Both predictor and behaviour/outcome reported at the within-person level	Either the predictor or the behaviour/outcome aggregated to the between-person level	Both predictor and behaviour/outcome aggregated to the between-person level

Table 1. Quality appraisal in included EMA studies.

Data synthesis

A narrative (descriptive) synthesis will be conducted. We will summarise the number of EMA studies conducted for each of the five health behaviours, study setting (e.g., country, immediate study setting), and sample size (i.e. mean or median number of participants per study). We will then present results in relation to each research question. First, we will

For peer review only - http://bmjopen.bmj.com/site/about/guidelines.xhtml

summarise study and EMA characteristics, e.g., study setting, population characteristics, percentage prompting frequency (e.g., % daily, % weekly), percentage type of EMA method (e.g., % event contingent, % random assessments, % continuous sensor based, % hybrid), percentage type of EMA delivery mode (e.g., % smartphone app delivery), percentage type of incentive structure (e.g., % flat payment, % payment per EMA, % no incentive), rates of EMA adherence (mean or median), and study duration (mean or median). Second, we will summarise within- and between-person predictor-behaviour and predictor-outcome associations across EMA studies (e.g., the type of psychological predictor/outcome assessed, measurement type, frequency of measurement). We will then assess, with regression analyses, whether EMA adherence varies depending on study setting, study characteristics, participant characteristics, or type of incentive schedule used. We do not have any pre-specified hypotheses. Exploratory analyses will be conducted in R v.3.5.1. If there is sufficient homogeneity between studies (e.g., similar predictors/outcomes assessed with similar measurement type and frequency), within- or between-person predictor-behaviour or predictor-outcome associations (e.g., odds ratios, relative risks, correlation coefficients, regression coefficients) will be synthesised with random effects meta-analyses, grouped by behaviour. Analyses will be conducted with the 'metafor' or ‘CTmeta’ packages (30–32), as appropriate, also utilising ‘jamovi’ (33). Where means, standard deviations, etc., are not reported in the publications, we may contact study authors to request access to additional information.

Patient and public involvement

A patient and public involvement representative reviewed a lay summary of the protocol for our systematic review. Positive feedback was received on the review’s aims, the importance of the current research and choice of key behaviours relevant to public health. Once the review is completed, feedback will be sought from the additional patient and public involvement representatives about the interpretation of findings and plans for dissemination.

We will seek advice on how to best present the study outcomes and use them in order to design studies and interventions that are useful and relevant for the public.

ETHICS AND DISSEMINATION

This study does not require ethics approval as it will summarise data from previously published studies. A protocol was pre-registered on the international Prospective Register of Systematic Reviews (PROSPERO) and on the Open Science Framework; it will also be offered for peer-review and publication in an open access journal. The findings of the review will be disseminated through peer-reviewed publications and presentations at relevant conferences. The dataset will be made available to other researchers online via the creation of a digital object identifier, thus enabling further research questions to be addressed. We expect this review to be useful for researchers and healthcare practitioners who regularly design and interpret results from EMA studies. In the event that the number of studies identified is deemed too large to comprehensively describe all studies in one review article, additional topic- or behaviour-specific articles may be written.

Summary

EMA is a frequently used research method; however, an overview of studies using this method across key public health behaviours in healthy adults is lacking. This review will provide a comprehensive overview of associations between a psychological/contextual predictor and a health behaviour/behavioural outcome in EMA studies focusing on physical activity and sedentary behaviour, dietary behaviours, alcohol consumption, tobacco smoking and sexual health behaviours. This review will inform the future design of EMA studies and it will influence practice of assessing individuals in real life settings and providing interventions that are delivered at the time and place when and where required. This review will set a blueprint for how to conduct EMA studies to improve participants' adherence, participant burden and conduct meaningful studies in real life settings.

For peer review only

REFERENCES

1. Stone AA, Shiffman S. Ecological momentary assessment (EMA) in behavorial medicine. Ann Behav Med. 1994;
2. Johnston DW, Johnston M. Useful theories should apply to individuals. Br J Health Psychol. 2013;18(3):469–73.
3. Santangelo P, Bohus M, Ebner-Priemer UW. Ecological momentary assessment in borderline personality disorder: a review of recent findings and methodological challenges. J Personal Disord. 2014;28(4):555–76.
4. Bell IH, Lim MH, Rossell SL, Thomas N. Ecological momentary assessment and intervention in the treatment of psychotic disorders: a systematic review. Psychiatr Serv. 2017;68(11):1172–81.
5. aan het Rot M, Hogenelst K, Schoevers RA. Mood disorders in everyday life: A systematic review of experience sampling and ecological momentary assessment studies. Clin Psychol Rev. 2012;32(6):510–23.
6. Haedt-Matt AA, Keel PK. Revisiting the affect regulation model of binge eating: a meta-analysis of studies using ecological momentary assessment. Psychol Bull. 2011;137(4):660.
7. Goldschmidt AB, Wonderlich SA, Crosby RD, Engel SG, Lavender JM, Peterson CB, et al. Ecological momentary assessment of stressful events and negative affect in bulimia nervosa. J Consult Clin Psychol. 2014;82(1):30.
8. Walz LC, Nauta MH, aan het Rot M. Experience sampling and ecological momentary assessment for studying the daily lives of patients with anxiety disorders: A systematic review. J Anxiety Disord. 2014;28(8):925–37.
9. Mote J, Fulford D. Ecological momentary assessment of everyday social experiences of people with schizophrenia: A systematic review. Schizophr Res. 2020;216:56–68.
10. Morgenstern J, Kuerbis A, Muench F. Ecological momentary assessment and alcohol use disorder treatment. Alcohol Res Curr Rev. 2014;36(1):101.
11. May M, Junghaenel DU, Ono M, Stone AA, Schneider S. Ecological momentary assessment methodology in chronic pain research: a systematic review. J Pain. 2018;19(7):699–716.
12. Wen CKF, Schneider S, Stone AA, Spruijt-Metz D. Compliance with mobile ecological momentary assessment protocols in children and adolescents: a systematic review and meta-analysis. J Med Internet Res. 2017;19(4):e132.
13. Heron KE, Everhart RS, McHale SM, Smyth JM. Using mobile-technology-based ecological momentary assessment (EMA) methods with youth: A systematic review and recommendations. J Pediatr Psychol. 2017;42(10):1087–107.
14. Cain AE, Depp CA, Jeste DV. Ecological momentary assessment in aging research: a critical review. J Psychiatr Res. 2009;43(11):987–96.

For peer review only

15. Dunton GF. Ecological momentary assessment in physical activity research. Exerc Sport Sci Rev. 2017;45(1):48.

16. Degroote L, DeSmet A, De Bourdeaudhuij I, Van Dyck D, Crombez G. Content validity and methodological considerations in ecological momentary assessment studies on physical activity and sedentary behaviour: a systematic review. Int J Behav Nutr Phys Act. 2020;17(1):1–13.

17. Wray TB, Merrill JE, Monti PM. Using ecological momentary assessment (EMA) to assess situation-level predictors of alcohol use and alcohol-related consequences. Alcohol Res Curr Rev. 2014;36(1):19.

18. Serre F, Fatseas M, Swendsen J, Auriacombe M. Ecological momentary assessment in the investigation of craving and substance use in daily life: a systematic review. Drug Alcohol Depend. 2015;148:1–20.

19. Maugeri A, Barchitta M. A systematic review of ecological momentary assessment of diet: implications and perspectives for nutritional epidemiology. Nutrients. 2019;11(11):2696.

20. Scott-Sheldon LA, Carey KB, Cunningham K, Johnson BT, Carey MP, MASH Research Team. Alcohol use predicts sexual decision-making: a systematic review and meta-analysis of the experimental literature. AIDS Behav. 2016;20(1):19–39.

21. Liu H, Xie QW, Lou VW. Everyday social interactions and intra-individual variability in affect: A systematic review and meta-analysis of ecological momentary assessment studies. Motiv Emot. 2019;43(2):339–53.

22. Schembre SM, Liao Y, O'connor SG, Hingle MD, Shen S-E, Hamoy KG, et al. Mobile ecological momentary diet assessment methods for behavioral research: Systematic review. JMIR MHealth UHealth. 2018;6(11):e11170.

23. Jones A, Remmerswaal D, Verveer I, Robinson E, Franken IHA, Wen CKF, et al. Compliance with ecological momentary assessment protocols in substance users: a meta-analysis. Addiction. 2019 Apr;114(4):609–19.

24. Collins LM. Analysis of longitudinal data: The integration of theoretical model, temporal design, and statistical model. Annu Rev Psychol. 2006;57:505–28.

25. WHO. Obesity and overweight [Internet]. 2018 [cited 2020 Jan 11]. Available from: https://www.who.int/news-room/fact-sheets/detail/obesity-and-overweight

26. Veritas Health Innovation. Covidence systematic review software. 2016;

27. Liao Y, Skelton K, Dunton G, Bruening M. A Systematic Review of Methods and Procedures Used in Ecological Momentary Assessments of Diet and Physical Activity Research in Youth: An Adapted STROBE Checklist for Reporting EMA Studies (CREMAS). J Med Internet Res. 2016 Jun 21;18(6):e151.

28. Taylor PJ, Kolt GS, Vandelanotte C, Caperchione CM, Mummery WK, George ES, et al. A review of the nature and effectiveness of nutrition interventions in adult males – a guide for intervention strategies. Int J Behav Nutr Phys Act. 2013;10(1):13.

For peer review only - http://bmjopen.bmj.com/site/about/guidelines.xhtml

29. Thomas BH, Ciliska D, Dobbins M, Micucci S. A Process for Systematically Reviewing the Literature: Providing the Research Evidence for Public Health Nursing Interventions. Worldviews Evid Based Nurs. 2004 Sep;1(3):176–84.

30. Balaji Y, Sankaranarayanan S, Chellappa R. Metareg: Towards domain generalization using meta-regularization. In 2018. p. 998–1008.

31. Viechtbauer W. Metafor: meta-analysis package for R. R Package Version. 2010;2010:1–0.

32. Kuiper RM, Ryan O. Meta-analysis of Lagged Regression Models: A Continuous-time Approach. Struct Equ Model Multidiscip J. 2020 May 3;27(3):396–413.

33. Sahin MD, Aybek EC. Jamovi: An Easy to Use Statistical Software for the Social Scientists. Int J Assess Tools Educ. 2019;6(4):670–92.

For peer review only

Authors' contributions

DK, OP, DP and FN conceived the project. DK and OP are the project leads and coordinators, they jointly drafted the manuscript. All authors have made conceptual contributions to project design and procedures. All authors read, edited and approved the final version.

Funding

Dominika Kwasnicka's work is carried out within the HOMING program of the Foundation for Polish Science co-financed by the European Union under the European Regional Development Fund; grant number POIR.04.04.00-00-5CF3/18-00; HOMING 5/2018. Dimitra Kale and Olga Perski receive salary support from Cancer Research UK (C1417/A22962). Daniel Powell is funded by the Scottish Government's Rural and Environment Science and Analytical Services (RESAS) and by the School of Medicine, Medical Sciences, and Nutrition (SMMSN) at the University of Aberdeen. Felix Naughton's salary is covered by the Faculty of Medicine and Health Sciences at the University of East Anglia.

Acknowledgments

The review team would like to thank Dr Marta Marques for comments and suggestions on the initial version of the review protocol. The authors would like to thank the patient and public involvement representative who commented on the lay summary of our proposed plan, for their contribution to this research.

Competing interests

The authors have no competing interests to declare.

Data statement

Data associated with this manuscript will be available on OSF.

For peer review only

Supplementary Material 1

PRISMA-P (Preferred Reporting Items for Systematic review and Meta-Analysis Protocols)

2015 checklist: recommended items to address in a systematic review protocol*

Section and topic	Item No	Checklist item	Page number
**ADMINISTRATIVE INFORMATION**			
Title:			
Identification	1a	Identify the report as a protocol of a systematic review	1
Update	1b	If the protocol is for an update of a previous systematic review, identify as such	NA
Registration	2	If registered, provide the name of the registry (such as PROSPERO) and registration number	3
Authors:			
Contact	3a	Provide name, institutional affiliation, e-mail address of all protocol authors; provide physical mailing address of corresponding author	1-2
Contributions	3b	Describe contributions of protocol authors and identify the guarantor of the review	18
Amendments	4	If the protocol represents an amendment of a previously completed or published protocol, identify as such and list changes; otherwise, state plan for documenting important protocol amendments	NA
Support:			
Sources	5a	Indicate sources of financial or other support for the review	18
Sponsor	5b	Provide name for the review funder and/or sponsor	18
Role of sponsor or funder	5c	Describe roles of funder(s), sponsor(s), and/or institution(s), if any, in developing the protocol	18
**INTRODUCTION**			
Rationale	6	Describe the rationale for the review in the context of what is already known	6
Objectives	7	Provide an explicit statement of the question(s) the review will address with reference to participants, interventions, comparators, and outcomes (PICO)	6-7
**METHODS**			
Eligibility criteria	8	Specify the study characteristics (such as PICO, study design, setting, time frame) and report characteristics (such as years considered, language, publication status) to be used as criteria for eligibility for the review	7-8

For peer review only

Information sources	9	Describe all intended information sources (such as electronic databases, contact with study authors, trial registers or other grey literature sources) with planned dates of coverage	8-9
Search strategy	10	Present draft of search strategy to be used for at least one electronic database, including planned limits, such that it could be repeated	9; Appendix 2
Study records:			
Data management	11a	Describe the mechanism(s) that will be used to manage records and data throughout the review	9-11
Selection process	11b	State the process that will be used for selecting studies (such as two independent reviewers) through each phase of the review (that is, screening, eligibility and inclusion in meta-analysis)	9-11
Data collection process	11c	Describe planned method of extracting data from reports (such as piloting forms, done independently, in duplicate), any processes for obtaining and confirming data from investigators	10-11
Data items	12	List and define all variables for which data will be sought (such as PICO items, funding sources), any pre-planned data assumptions and simplifications	10-11
Outcomes and prioritization	13	List and define all outcomes for which data will be sought, including prioritization of main and additional outcomes, with rationale	10-11
Risk of bias in individual studies	14	Describe anticipated methods for assessing risk of bias of individual studies, including whether this will be done at the outcome or study level, or both; state how this information will be used in data synthesis	11-12
Data synthesis	15a	Describe criteria under which study data will be quantitatively synthesised	12-13
	15b	If data are appropriate for quantitative synthesis, describe planned summary measures, methods of handling data and methods of combining data from studies, including any planned exploration of consistency (such as I^2, Kendall's τ)	13
	15c	Describe any proposed additional analyses (such as sensitivity or subgroup analyses, meta-regression)	13
	15d	If quantitative synthesis is not appropriate, describe the type of summary planned	13
Meta-bias(es)	16	Specify any planned assessment of meta-bias(es) (such as publication bias across studies, selective reporting within studies)	11-12
Confidence in cumulative evidence	17	Describe how the strength of the body of evidence will be assessed (such as GRADE)	11-12

* It is strongly recommended that this checklist be read in conjunction with the PRISMA-P Explanation and Elaboration (cite when available) for important clarification on the items. Amendments to a review protocol should be tracked and dated. The copyright for PRISMA-P

For peer review only

(including checklist) is held by the PRISMA-P Group and is distributed under a Creative Commons Attribution Licence 4.0. *From: Shamseer L, Moher D, Clarke M, Ghersi D, Liberati A, Petticrew M, Shekelle P, Stewart L, PRISMA-P Group. Preferred reporting items for systematic review and meta-analysis protocols (PRISMA-P) 2015: elaboration and explanation. BMJ. 2015 Jan 2;349(jan02 1):g7647.*

For peer review only

For peer review only

For peer review only - http://bmjopen.bmj.com/site/about/guidelines.xhtml

Supplementary Material 2

Full search strategy: **Ovid MEDLINE, Embase, PsycINFO**

1. (ecological adj1 momentary adj1 assessment*).ti,ab.

2. (intensive adj1 longitudinal).ti,ab.

3. (ambulatory adj1 assessment*).ti,ab.

4. (experience adj1 sampl*).ti,ab.

5. (daily adj1 diar*).ti,ab.

6. (ecological adj1 momentary adj1 intervention).ti,ab.

7. within-person.ti,ab.

8. within-subject*.ti,ab.

9. (single adj1 case).ti,ab.

10. idiographic.ti,ab.

11. 1 OR 2 OR 3 OR 4 OR 5 OR 6 OR 7 OR 8 OR 9 OR 10

12. tobacco.ti,ab.

13. smok*.ti,ab.

14. alcohol*.ti,ab.

15. diet.ti,ab.

16. weight.ti,ab.

17. overweight.ti,ab.

18. obes*.ti,ab.

19. (healthy adj3 eat*).ti,ab.

20. physical activity.ti,ab.

21. exercise.ti,ab.

22. (sexual adj1 health).ti,ab.

23. condom.ti,ab.

For peer review only

24. contraceptive.ti,ab.

25. 12 OR 13 OR 14 OR 15 OR 16 OR 17 OR 18 OR 19 OR 20 OR 21 OR 22 OR 23 OR 24

26. 11 AND 25

Results: **12,677**

Web of Science

1. TS=(ecological NEAR/1 momentary NEAR/1 assessment*)

2. TS=(ecological NEAR/1 momentary NEAR/1 intervention)

3. TS=(intensive NEAR/1 longitudinal)

4. TS=(ambulatory NEAR/1 assessment*)

5. TS=(experience NEAR/1 sampl*)

6. TS=(daily NEAR/1 diar*)

7. TS=(within-person or within-subject* or idiographic)

8. TS=(single NEAR/1 case)

9. 1 OR 2 OR 3 OR 4 OR 5 OR 6 OR 7 OR 8

10. TS=(healthy NEAR/1 eat*)

11. TS=(sexual NEAR/1 health)

12. TS=(smok* or tobacco* or alcohol* or diet or weight or overweight or obes* or physical activity or exercise or condom or contraceptive)

13. 10 OR 11 OR 12

14. 9 AND 13

Results: **8,141**

For peer review only

Systematic review of Ecological Momentary Assessment (EMA) studies of five public health-related behaviours: Review Protocol.

Journal:	*BMJ Open*
Manuscript ID	bmjopen-2020-046435.R1
Article Type:	Protocol
Date Submitted by the Author:	31-Mar-2021
Complete List of Authors:	Kwasnicka, Dominika; SWPS University of Social Sciences and Humanities Wroclaw Campus Library, Faculty of Psychology<br>Kale, Dimitra; University College London<br>Schneider, Verena; University College London<br>Keller, Jan; Freie Universität Berlin<br>Yeboah-Asiamah Asare, Bernard; Curtin University<br>Powell, Daniel; University of Aberdeen<br>Naughton, Felix; University of East Anglia Faculty of Medicine and Health Sciences, School of Health Sciences; University of East Anglia<br>ten Hoor, Gill A. ; Department of Work & Social Psychology, Maastricht University,<br>Verboon, Peter; Open Universiteit Nederland Faculteit Managementwetenschappen<br>Perski, Olga; University College London, Department of Behavioural Science and Health
**Primary Subject Heading**:	Public health
Secondary Subject Heading:	Public health
Keywords:	PUBLIC HEALTH, STATISTICS & RESEARCH METHODS, SOCIAL MEDICINE, SPORTS MEDICINE, NUTRITION & DIETETICS

SCHOLARONE™
Manuscripts

I, the Submitting Author has the right to grant and does grant on behalf of all authors of the Work (as defined in the below author licence), an exclusive licence and/or a non-exclusive licence for contributions from authors who are: i) UK Crown employees; ii) where BMJ has agreed a CC-BY licence shall apply, and/or iii) in accordance with the terms applicable for US Federal Government officers or employees acting as part of their official duties; on a worldwide, perpetual, irrevocable, royalty-free basis to BMJ Publishing Group Ltd ("BMJ") its licensees and where the relevant Journal is co-owned by BMJ to the co-owners of the Journal, to publish the Work in this journal and any other BMJ products and to exploit all rights, as set out in our licence.

The Submitting Author accepts and understands that any supply made under these terms is made by BMJ to the Submitting Author unless you are acting as an employee on behalf of your employer or a postgraduate student of an affiliated institution which is paying any applicable article publishing charge ("APC") for Open Access articles. Where the Submitting Author wishes to make the Work available on an Open Access basis (and intends to pay the relevant APC), the terms of reuse of such Open Access shall be governed by a Creative Commons licence – details of these licences and which Creative Commons licence will apply to this Work are set out in our licence referred to above.

Other than as permitted in any relevant BMJ Author's Self Archiving Policies, I confirm this Work has not been accepted for publication elsewhere, is not being considered for publication elsewhere and does not duplicate material already published. I confirm all authors consent to publication of this Work and authorise the granting of this licence.

For peer review only

Title: Systematic review of Ecological Momentary Assessment (EMA) studies of five public health-related behaviours: Review Protocol.

Authors: Dominika Kwasnicka[1,2], Dimitra Kale[3], Verena Schneider[3], Jan Keller[4], Bernard Yeboah-Asiamah Asare[5,6], Daniel Powell[6,7], Felix Naughton[8], Gill ten Hoor[9], Peter Verboon[10], Olga Perski[3]

1. Faculty of Psychology, SWPS University of Social Sciences and Humanities, Aleksandra Ostrowskiego 30b, 53-238, Wroclaw, Poland

2. NHMRC CRE in Digital Technology to Transform Chronic Disease Outcomes, Melbourne School of Population and Global Health, University of Melbourne, 333 Exhibition Street, 3000, Melbourne, Australia

3. Department of Behavioural Science and Health, Institute of Epidemiology and Health, Faculty of Population Health Sciences, University College London, United Kingdom

4. Department of Education and Psychology, Freie Universität Berlin, Berlin, Germany

5. School of Public Health, Curtin University, Kent Street, 6102, Perth, Australia

6. Health Psychology, Institute of Applied Health Sciences, University of Aberdeen, Aberdeen, AB25 2ZD, United Kingdom

7. Rowett Institute, University of Aberdeen, Aberdeen, AB25 2ZD, United Kingdom

8. Behavioural and Implementation Science Research Group, School of Health Sciences, University of East Anglia, Norwich, NR4 7UL, United Kingdom

9. Department of Work and Social Psychology, Faculty of Psychology and Neurosciences, Maastricht University, Maastricht, The Netherlands

For peer review only

10. Faculty of Psychology, Open University, Heerlen, The Netherlands

Corresponding author: Dominika Kwasnicka, Faculty of psychology SWPS University of Social Sciences and Humanities, Aleksandra Ostrowskiego 30b, 53-238, Wrocław and NHMRC CRE in Digital Technology to Transform Chronic Disease Outcomes, Melbourne School of Population and Global Health, University of Melbourne, 333 Exhibition Street, 3000, Melbourne, Australia. E-mail: dkwasnicka@swps.edu.pl

Word count: 3,446

Keywords: ambulatory assessment, Ecological Momentary Assessment, EMA, experience sampling, health, psychology, systematic review, within-person design

For peer review only

ABSTRACT

Introduction: Ecological Momentary Assessment (EMA) involves repeated, real-time assessments of phenomena (e.g., cognitions, emotions, behaviours) over a period of time in naturalistic settings. EMA is increasingly used to study both within- and between-person processes. We will review EMA studies investigating key health behaviours and synthesise: 1) study characteristics (e.g., frequency of assessments, adherence, incentives), 2) associations between psychological predictors and behaviours, and 3) moderators of adherence to EMA protocols.

Methods and analysis: This review will focus on EMA studies conducted across five public health behaviours in adult, non-clinical populations: movement behaviour (including physical activity and sedentary behaviour), dietary behaviour, alcohol consumption, tobacco smoking, and preventive sexual health behaviours. Studies need to have assessed at least one psychological or contextual predictor of these behaviours. Studies reporting exclusively on physiological outcomes (e.g., cortisol) or those not conducted under free-living conditions will be excluded. We will search OVID Medline, Embase, PsycINFO and Web of Science using terms relevant to EMA and the selected health behaviours. Reference lists of existing systematic reviews of EMA studies will be hand searched. Identified articles will be screened by two reviewers. This review is expected to provide a comprehensive summary of EMA studies assessing psychological or contextual predictors of five public health behaviours.

Ethics and dissemination: The results will be disseminated through peer-reviewed publications and presentations. Data from included studies will be made available to other researchers. No ethics are required.

Review registration: The review protocol has been registered with PROSPERO 2020 CRD42020168314. Available from: www.crd.york.ac.uk/prospero/display_record.php?ID=CRD42020168314.

For peer review only - http://bmjopen.bmj.com/site/about/guidelines.xhtml

Strengths and limitations of this study:

- A protocol for systematic review is provided for EMA studies in adult, non-clinical populations.
- We included EMA studies of five key public health behaviours including movement behaviours, dietary behaviours, alcohol consumption, tobacco smoking, and preventive sexual health behaviours.
- We will review characteristics of EMA studies (e.g., study duration in days, incentives, adherence rates) and associations between psychological predictors and behaviours, examining rates of adherence to EMA protocols across different health behaviours and moderators of adherence (e.g., study setting, type of behaviour).
- Extracted data will be made available to other researchers, thus allowing for the exploration of additional research questions and potential for setting up a 'living review'.
- As included studies are likely to be heterogeneous, this may limit the overarching conclusions that can be drawn, and will likely prevent meta-analysis combining effect sizes from multiple studies and across all behaviours.

For peer review only - http://bmjopen.bmj.com/site/about/guidelines.xhtml

INTRODUCTION

Ecological Momentary Assessment (EMA), also known as ambulatory assessment or experience sampling methodology, involves repeated, real-time assessments of phenomena (e.g., cognitions, emotions and behaviours) over a period of time in naturalistic settings (1). EMA is increasingly used to study within- and between-person processes, including associations between psychological and health behaviour-related variables (e.g., positive affect and physical activity in general population or stress and lapse risk in smokers attempting to stop). For researchers and healthcare professionals to understand and change behaviour, it is important for theories and interventions to be applicable to both momentary states of individuals (within-person processes) and groups of individuals (between-person processes) (2). Despite the popularity and importance of EMA for studying health-related behaviours, there has been no comprehensive systematic investigation of characteristics of EMA studies (e.g., rates of adherence, study duration in days, incentive schedules) and potential moderators of adherence (e.g., study setting, type of health behaviour), with attempts to describe associations between psychological predictors (e.g., intentions, self-efficacy) and key public health behaviours.

Previous reviews of EMA studies have focused on clinical conditions such as borderline personality disorder (3), psychotic disorder (4), mood disorders (5), binge eating (6), bulimia nervosa (7), anxiety disorder (8), schizophrenia (9), alcohol use disorder (10), chronic pain (11), and specific populations such as children and adolescents (12), youth (13) and older adults (14). Health behaviour-specific reviews of EMA studies have focused on physical activity (15,16), sedentary behaviour (16), alcohol use (17), craving and substance use (18), dietary behaviours (19), and the relationship between alcohol use and sexual decision making (20). Previous EMA reviews have also focused on interrelations between specific psychological

For peer review only

variables, such as the association of everyday social interactions with intra-individual variability in affect (21).

While systematic reviews of EMA studies focusing on specific health behaviours have been conducted (18,22,23), there are no overarching reviews that can help address broad questions about study characteristics (e.g., study duration in days, adherence, incentive schedules), rates and moderators of adherence (e.g., study setting, participant characteristics) and predictor-behaviour associations across different health behaviours and potential moderators (e.g., study setting, study quality). It is expected that this review will help fill this gap. We also expect that this review will help inform the design of future EMA studies by providing a summary of best practice across research contexts, settings and health-related behaviours. For instance, results may be useful for informing researchers' understanding of what frequency or intensity of change we would expect to see at what temporal resolution [i.e., informed by a 'theory of change' (24)], which can then inform assessment scheduling decisions. This review is likely to include a large number of studies, thus providing a comprehensive overview of the EMA literature.

The current study

We will synthesise evidence from EMA studies that report either within- or between-person predictor-behaviour associations. The review will focus on five key public health behaviours: (1) movement behaviours (including physical activity and sedentary behaviour), (2) dietary behaviours, (3) alcohol consumption, (4) tobacco smoking and (5) preventive sexual health behaviours (including contraceptive use).

The review aims are:

1. To summarise adherence to EMAs, total length of data collection of EMAs, prompting frequency of EMAs, and incentives structures across studies.

For peer review only

2. To describe within- and between-person predictor-behaviour associations across EMA studies (e.g., associations between intention and behaviour).
3. To assess potential moderators of adherence to EMAs (e.g., study setting, participant characteristics).

This review is intentionally broad in scope to provide an overview of the field for researchers interested in the application of EMAs to the study of health-related behaviours. We expect this overarching review to help identify patterns and key knowledge gaps.

METHODS AND ANALYSIS

Study design

This review will adhere to the Preferred Reporting Items for Systematic Reviews and Meta-Analyses (PRISMA) checklist (Supplementary Material 1). The review start date was 15th September 2019 and the planned end date is 30th December 2021.

Inclusion criteria

This review will focus on five key public health behaviours in healthy adults (i.e. non-clinical populations) aged 18+ years, namely:

1. movement behaviours, including physical activity and sedentary behaviour;
2. dietary behaviours, including snacking or fruit and vegetable consumption;
3. alcohol consumption;
4. tobacco smoking, including cigarette, cigar or pipe smoking;
5. preventive sexual health behaviours, including contraceptive/condom use.

No restrictions on geographical location or publication date will be set. To be included, studies need to incorporate multiple (i.e., two or more) within-day, daily or weekly assessments of predictors and behaviours, and to have reported either (or both) within- or between-person predictor-behaviour (e.g., stress predicting unhealthy snack consumption) associations. The

For peer review only

frequency of the EMAs should plausibly match how the target behaviour (and psychological and contextual predictors) theoretically or empirically unfolds over time, e.g., daily assessments of steps, weekly assessments of gym class attendance if the class is undertaken only once a week. To be included, studies need to assess one of the aforementioned behaviours and at least one psychological or contextual variable via EMAs.

In this review, we defined psychological variables as emergent properties of a distributed network of neurons, including cognition (e.g., beliefs, attitudes, goals), emotion (e.g., negative affect, cravings) and processes operating on these (e.g., self-regulation, learning), which are linked to behaviour. We further define contextual variables as any potential environmental (e.g., social or physical) influences on behaviour, including the presence of other people, weather, or the availability of unhealthy foods/cigarettes/alcohol. The psychological and contextual variables will be closely assessed by the reviewers as to their suitability for inclusion/exclusion in the review.

In addition to self-report measures, included studies can use physiological measures of psychological predictors (e.g., cortisol or heart rate variability to measure stress) or behaviours (e.g., accelerometer data to measure physical activity or sedentary behaviour). Studies reporting associations between behaviours and psychological consequences (e.g., whether physical activity predicts affect) will be included providing that they also report psychological or contextual predictor-behaviour associations (e.g., whether positive affect predicts physical activity). We will include individuals with overweight and obesity given that 39% of adults globally fall into this category, with most Western countries averaging above 50% (25). Studies including participants with a diagnosed mental or physical health condition who were not recruited into the study on the basis of their condition will be included (e.g., studies including participants with clinical levels of depression but where this was not an inclusion criterion).

For peer review only - http://bmjopen.bmj.com/site/about/guidelines.xhtml

Studies in which a behavioural or pharmacological intervention was delivered will be included providing that participants were asked to complete free-living EMAs.

Exclusion criteria

Laboratory studies will not be included. Studies examining clinical populations, that is, solely recruiting participants on the basis of being diagnosed with a physical or mental health condition such as cancer, cardiovascular disease, depression, binge eating disorder or substance use disorder (also including case-control studies) will be excluded. Studies focusing only on purchasing behaviours (e.g., tobacco purchasing, food purchasing) will not be included. Studies focusing on e-cigarettes will be also excluded. Studies not published in English or where no full text could be obtained will also not be included. Although behaviour-behaviour associations may also be considered relevant, our electronic search is not designed to capture such studies, and behaviour-behaviour associations will hence not be considered further in this review.

Search methods for the identification of studies

Electronic searches

We will search Ovid MEDLINE, Embase, PsycINFO and Web of Science (see Supplementary Material 2 for the full search strategy). Terms will be searched in titles and abstracts as free text terms or as index terms (e.g., Medical Subject Headings), as appropriate. We will combine two groups of terms, the first with terms relevant to EMAs and within-person study designs; the second with terms relevant to the five health behaviours addressed in this review.

Example terms used:

1. (ecological adj1 momentary adj1 assessment*) OR (intensive adj1 longitudinal) OR (ambulatory adj1 assessment*) OR (experience adj1 sampl*) OR (daily adj1 diar*) OR (ecological adj1 momentary adj1 intervention) OR within-person OR within-subject* OR (single adj1 case) OR idiographic OR intraindividual

For peer review only - http://bmjopen.bmj.com/site/about/guidelines.xhtml

2. tobacco OR smok* OR cigarette OR alcohol* OR drinking OR addict* OR (healthy adj3 eat*) OR diet OR weight OR overweight OR obes* OR physical activity OR exercise OR sedentary OR sitting OR leisure OR (sexual adj1 health) OR condom OR contraceptive
3. 1 AND 2

Electronic and hand searches were conducted in January 2020 and updated in February 2021. We restricted the search to human studies available in English that are published in peer-reviewed journals (Online Attachment 2).

Searching for other sources

Reference lists of existing systematic reviews of EMA studies will be hand searched and expertise within the review team will be used to identify additional articles of interest.

Data collection and analysis

Selection of studies

Identified articles will be merged using Covidence (26) and duplicate records will be removed. The three lead authors (DK, OP and JK) will independently screen titles and abstracts (yes, maybe, no) against the pre-specified inclusion criteria. Full texts will be screened by two reviewers independently (yes, no); discrepancies will be resolved by the lead authors and inclusion will be further discussed with other team members if needed. In line with the PRISMA checklist, key reasons for exclusion will be recorded at the full text stage. These will include: lack of psychological predictors or outcomes; study not being relevant to the five key public health behaviours of interest; wrong study design (not an EMA study); participants being recruited based on a health condition (i.e., clinical population); participants younger than 18 years old; studies of purchasing behaviours; conference abstracts; protocols; duplicates; studies not published in English or no full text could be obtained. We will follow the hierarchy

For peer review only

of the exclusion criteria, listing the first reason from the aforementioned list as the key reason for exclusion.

Data extraction and management

A data extraction form will be developed in Microsoft Excel to extract information and to import data into R for analysis. Each study will be allocated a unique study identification number. Data will be extracted on:

- *Study description* (study author, year, country, study funder);
- *Participant characteristics* (sample size; mean or median age (SD); gender (% female); educational attainment (% university education); population type (e.g., men who have sex with men, older adults, general population), ethnicity (% White ethnicity);
- *EMA study type* (e.g., observational, interventional, both);
- *EMA delivery mode* (e.g., mobile phone, website/online, pen-and-paper);
- *EMA method* (e.g., signal contingent, event contingent, multiple);
- *EMA characteristics* (e.g., total study duration in days; prompting frequency (e.g., hourly, daily, weekly), incentive schedule (e.g., flat rate, payment per EMA);
- *Adherence to EMA* (e.g., average % EMAs completed out of available prompts);
- *Health behaviour(s) assessed* (e.g., physical activity, sedentary behaviour, dietary behaviour, tobacco smoking); and how the health behaviour(s) were measured (e.g., hourly step count, number of cigarettes smoked per day);
- *Psychological and contextual predictors* (e.g., intentions, self-efficacy, presence of other smokers) and how they were measured (e.g., EMA method, measurement frequency, whether the measure was developed for the study (versus precedent), whether a single item or multiple items were used);

For peer review only - http://bmjopen.bmj.com/site/about/guidelines.xhtml

- *Statistical model used to examine predictor-behaviour association* (e.g. multilevel model, generalised estimating equation) and whether these associations were analysed on the within- and/or between-level;
- *Level of aggregation in data analysis* (i.e. whether data underpinning the predictor-behaviour association are aggregated vs. maintained at the within-person level);
- *Coefficients and effect sizes from statistical models* (e.g. odds ratios, relative risks, regression coefficients);
- *Control variables in multivariate models* (e.g. age, sex)

For each study, one reviewer will extract the data. At least 20% of studies stratified by behaviour (e.g., 20% of all alcohol consumption studies) will be double checked for accuracy and completeness by a second reviewer. In case there are any uncertainties related to data extraction (e.g., the primary data extractor is uncertain about a particular parameter or a large number of discrepancies are observed across the primary and secondary data extractor), we will double check additional studies until agreement is achieved. All review authors will be involved in data extraction and double checking.

Quality appraisal

Included studies may vary in quality, which will be considered through a quality appraisal. The appraisal tool was developed by the review team, based on an existing EMA reporting checklist (27), and includes the following four criteria: 1) rationale for EMA design, 2) a priori power analysis to determine sample size, 3) percentage adherence to the EMA protocol, and 4) treatment of missingness (Table 1). The quality indicators will be coded by one reviewer, with 20% or more double checked by a second reviewer. Discrepancies will be resolved through discussion among the lead authors. Where possible, each study quality indicator will be entered as a moderator of predictor-behaviour associations. As each criterion

For peer review only - http://bmjopen.bmj.com/site/about/guidelines.xhtml

refers to a different aspect of study quality, we will not summarise study quality, but will present how studies score on each selected dimension.

Topic: Factors	Strong	Moderate	Weak
**Rationale**			
1. Rationale for EMA design provided*:* Why was an EMA design chosen to examine the research question?	A strong rationale provided for the EMA design of predictor AND behaviour/ outcome	Rationale provided but not very strong for the EMA design of either the predictor OR behaviour/ outcome	No rationale for the EMA design regarding predictor and behaviour/ outcome
**Power analysis, sample size and participant adherence**			
2. Power analysis*:* A priori power analysis to determine sample size	An a priori power analysis is reported and the enrolled sample size met power analysis indication / OR: sufficient explanation as to why an a priori power analysis was not needed	An a priori power analysis is reported but sufficient sample size/number of observations was not achieved	No information about power analysis / OR: a post-hoc power analysis is reported
3. Adherence to EMA protocol: Percentage of answered EMA prompts across all participants for the main EMA study period	Percentage of answered EMA prompts >80%	Percentage of answered EMA prompts 60-79.99%	Percentage of answered EMA prompts less than 60%
**Data analysis**			
4. Treatment of missingness: Report whether study dropout or non-adherence to EMAs (e.g., missed prompts) are related to specific variables	Missing mechanisms/predictors are identified, reported and mitigated for if needed	Missing mechanisms/predictors are identified and reported but not mitigated for	Missing mechanisms/predictors are not identified or reported

Table 1. Quality appraisal in included EMA studies.

Data synthesis

All quantitative analyses will be conducted in R v.3.5.1. A narrative (descriptive) synthesis will be conducted. We will summarise the number of EMA studies conducted for

For peer review only - http://bmjopen.bmj.com/site/about/guidelines.xhtml

each of the five health behaviours, study setting (e.g., country, immediate study setting), and sample size (i.e. mean or median number of participants per study). We will then present results in relation to each research question.

To address the first aim, we will summarise study and EMA characteristics, e.g., study setting, population characteristics, percentage prompting frequency (e.g., % daily, % weekly), percentage type of EMA method (e.g., % event contingent, % random assessments, % continuous sensor based, % hybrid), percentage type of EMA delivery mode (e.g., % smartphone app delivery), percentage type of incentive structure (e.g., % flat payment, % payment per EMA, % no incentive), rates of EMA adherence (mean or median), and study duration (mean or median).

To address the second aim, we will summarise within- and between-person predictor-behaviour associations across EMA studies (e.g., the type of psychological or contextual predictor assessed, measurement type, frequency of measurement). If there is sufficient homogeneity between studies (e.g., similar predictors assessed with similar measurement type and frequency across ≥3 studies), within- or between-person predictor-behaviour associations (e.g., odds ratios, relative risks, regression coefficients) will be synthesised with random effects meta-analyses, grouped by behaviour. Analyses will be conducted with the 'metafor' or 'CTmeta' packages (30–32), as appropriate, also utilising 'jamovi' (33). Where sufficient detail on model parameter estimates is lacking in the publications, we may contact study authors to request access to additional information.

To address the third aim, we will assess, with regression analyses, whether EMA adherence varies depending on study setting, study characteristics, participant characteristics, or type of incentive schedule used. We do not have any pre-specified hypotheses. Where appropriate, moderator analyses will be conducted to examine whether predictor-behaviour

associations vary depending on study setting, study characteristics, participant characteristics, or type of incentive schedule used.

Patient and public involvement

A patient and public involvement representative reviewed a lay summary of the protocol for our systematic review. Positive feedback was received on the review's aims, the importance of the current research and choice of key behaviours relevant to public health. Once the review is completed, feedback will be sought from the additional patient and public involvement representatives about the interpretation of findings and plans for dissemination. We will seek advice on how to best present the study outcomes and use them in order to design studies and interventions that are useful and relevant for the public.

ETHICS AND DISSEMINATION

This study does not require ethics approval as it will summarise data from previously published studies. A protocol was pre-registered on the international Prospective Register of Systematic Reviews (PROSPERO) and on the Open Science Framework; it will also be offered for peer-review and publication in an open access journal. The findings of the review will be disseminated through peer-reviewed publications and presentations at relevant conferences. The dataset will be made available to other researchers online via the creation of a digital object identifier, thus enabling further research questions to be addressed. We expect this review to be useful for researchers and healthcare practitioners who regularly design and interpret results from EMA studies. We plan to publish overarching review and subsequently five behaviour-specific reviews that will provide a more in-depth synthesis of predictor-behaviour associations.

For peer review only

Summary

EMA is a frequently used research method; however, an overview of studies using this method across key public health behaviours in healthy adults is lacking. This review will

provide a comprehensive overview of associations between a psychological/contextual predictor and a health behaviour in EMA studies focusing on movement behaviours, dietary behaviours, alcohol consumption, tobacco smoking and sexual health behaviours. This review will inform the future design of EMA studies and it will influence practice of assessing individuals in real life settings and providing interventions that are delivered at the time and place when and where required. This review will set a blueprint for how to conduct EMA studies to improve participants' adherence and conduct meaningful studies in real life settings.

For peer review only

REFERENCES

1. Stone AA, Shiffman S. Ecological momentary assessment (EMA) in behavorial medicine. Ann Behav Med. 1994;

2. Johnston DW, Johnston M. Useful theories should apply to individuals. Br J Health Psychol. 2013;18(3):469–73.

3. Santangelo P, Bohus M, Ebner-Priemer UW. Ecological momentary assessment in borderline personality disorder: a review of recent findings and methodological challenges. J Personal Disord. 2014;28(4):555–76.

4. Bell IH, Lim MH, Rossell SL, Thomas N. Ecological momentary assessment and intervention in the treatment of psychotic disorders: a systematic review. Psychiatr Serv. 2017;68(11):1172–81.

5. aan het Rot M, Hogenelst K, Schoevers RA. Mood disorders in everyday life: A systematic review of experience sampling and ecological momentary assessment studies. Clin Psychol Rev. 2012;32(6):510–23.

6. Haedt-Matt AA, Keel PK. Revisiting the affect regulation model of binge eating: a meta-analysis of studies using ecological momentary assessment. Psychol Bull. 2011;137(4):660.

7. Goldschmidt AB, Wonderlich SA, Crosby RD, Engel SG, Lavender JM, Peterson CB, et al. Ecological momentary assessment of stressful events and negative affect in bulimia nervosa. J Consult Clin Psychol. 2014;82(1):30.

8. Walz LC, Nauta MH, aan het Rot M. Experience sampling and ecological momentary assessment for studying the daily lives of patients with anxiety disorders: A systematic review. J Anxiety Disord. 2014;28(8):925–37.

9. Mote J, Fulford D. Ecological momentary assessment of everyday social experiences of people with schizophrenia: A systematic review. Schizophr Res. 2020;216:56–68.

10. Morgenstern J, Kuerbis A, Muench F. Ecological momentary assessment and alcohol use disorder treatment. Alcohol Res Curr Rev. 2014;36(1):101.

11. May M, Junghaenel DU, Ono M, Stone AA, Schneider S. Ecological momentary assessment methodology in chronic pain research: a systematic review. J Pain. 2018;19(7):699–716.

12. Wen CKF, Schneider S, Stone AA, Spruijt-Metz D. Compliance with mobile ecological momentary assessment protocols in children and adolescents: a systematic review and meta-analysis. J Med Internet Res. 2017;19(4):e132.

13. Heron KE, Everhart RS, McHale SM, Smyth JM. Using mobile-technology-based ecological momentary assessment (EMA) methods with youth: A systematic review and recommendations. J Pediatr Psychol. 2017;42(10):1087–107.

14. Cain AE, Depp CA, Jeste DV. Ecological momentary assessment in aging research: a critical review. J Psychiatr Res. 2009;43(11):987–96.

For peer review only

15. Dunton GF. Ecological momentary assessment in physical activity research. Exerc Sport Sci Rev. 2017;45(1):48.

16. Degroote L, DeSmet A, De Bourdeaudhuij I, Van Dyck D, Crombez G. Content validity and methodological considerations in ecological momentary assessment studies on physical activity and sedentary behaviour: a systematic review. Int J Behav Nutr Phys Act. 2020;17(1):1–13.

17. Wray TB, Merrill JE, Monti PM. Using ecological momentary assessment (EMA) to assess situation-level predictors of alcohol use and alcohol-related consequences. Alcohol Res Curr Rev. 2014;36(1):19.

18. Serre F, Fatseas M, Swendsen J, Auriacombe M. Ecological momentary assessment in the investigation of craving and substance use in daily life: a systematic review. Drug Alcohol Depend. 2015;148:1–20.

19. Maugeri A, Barchitta M. A systematic review of ecological momentary assessment of diet: implications and perspectives for nutritional epidemiology. Nutrients. 2019;11(11):2696.

20. Scott-Sheldon LA, Carey KB, Cunningham K, Johnson BT, Carey MP, MASH Research Team. Alcohol use predicts sexual decision-making: a systematic review and meta-analysis of the experimental literature. AIDS Behav. 2016;20(1):19–39.

21. Liu H, Xie QW, Lou VW. Everyday social interactions and intra-individual variability in affect: A systematic review and meta-analysis of ecological momentary assessment studies. Motiv Emot. 2019;43(2):339–53.

22. Schembre SM, Liao Y, O'connor SG, Hingle MD, Shen S-E, Hamoy KG, et al. Mobile ecological momentary diet assessment methods for behavioral research: Systematic review. JMIR MHealth UHealth. 2018;6(11):e11170.

23. Jones A, Remmerswaal D, Verveer I, Robinson E, Franken IHA, Wen CKF, et al. Compliance with ecological momentary assessment protocols in substance users: a meta-analysis. Addiction. 2019 Apr;114(4):609–19.

24. Collins LM. Analysis of longitudinal data: The integration of theoretical model, temporal design, and statistical model. Annu Rev Psychol. 2006;57:505–28.

25. WHO. Obesity and overweight [Internet]. 2018 [cited 2020 Jan 11]. Available from: https://www.who.int/news-room/fact-sheets/detail/obesity-and-overweight

26. Veritas Health Innovation. Covidence systematic review software. 2016;

27. Liao Y, Skelton K, Dunton G, Bruening M. A Systematic Review of Methods and Procedures Used in Ecological Momentary Assessments of Diet and Physical Activity Research in Youth: An Adapted STROBE Checklist for Reporting EMA Studies (CREMAS). J Med Internet Res. 2016 Jun 21;18(6):e151.

28. Taylor PJ, Kolt GS, Vandelanotte C, Caperchione CM, Mummery WK, George ES, et al. A review of the nature and effectiveness of nutrition interventions in adult males – a guide for intervention strategies. Int J Behav Nutr Phys Act. 2013;10(1):13.

For peer review only - http://bmjopen.bmj.com/site/about/guidelines.xhtml

29. Thomas BH, Ciliska D, Dobbins M, Micucci S. A Process for Systematically Reviewing the Literature: Providing the Research Evidence for Public Health Nursing Interventions. Worldviews Evid Based Nurs. 2004 Sep;1(3):176–84.

30. Balaji Y, Sankaranarayanan S, Chellappa R. Metareg: Towards domain generalization using meta-regularization. In 2018. p. 998–1008.

31. Viechtbauer W. Metafor: meta-analysis package for R. R Package Version. 2010;2010:1–0.

32. Kuiper RM, Ryan O. Meta-analysis of Lagged Regression Models: A Continuous-time Approach. Struct Equ Model Multidiscip J. 2020 May 3;27(3):396–413.

33. Sahin MD, Aybek EC. Jamovi: An Easy to Use Statistical Software for the Social Scientists. Int J Assess Tools Educ. 2019;6(4):670–92.

For peer review only

Authors' contributions

DK, OP, DP and FN conceived the project. DK and OP are the project leads and coordinators, they jointly drafted the manuscript. All authors (DK, DK, VS, JK, BYAA, DP, FN, GH, PV, OP) have made conceptual contributions to project design and procedures. All authors read, edited and approved the final version.

Funding

Dominika Kwasnicka's work is carried out within the HOMING program of the Foundation for Polish Science co-financed by the European Union under the European Regional Development Fund; grant number POIR.04.04.00-00-5CF3/18-00; HOMING 5/2018. Dimitra Kale and Olga Perski receive salary support from Cancer Research UK (C1417/A22962). Daniel Powell is funded by the Scottish Government's Rural and Environment Science and Analytical Services (RESAS) and by the School of Medicine, Medical Sciences, and Nutrition (SMMSN) at the University of Aberdeen. Felix Naughton's salary is covered by the Faculty of Medicine and Health Sciences at the University of East Anglia.

Acknowledgments

The review team would like to thank Dr Marta Marques for comments and suggestions on the initial version of the review protocol. The authors would like to thank the patient and public involvement representative who commented on the lay summary of our proposed plan, for their contribution to this research.

Competing interests

The authors have no competing interests to declare.

Data statement

Data associated with this manuscript will be available on OSF.

For peer review only - http://bmjopen.bmj.com/site/about/guidelines.xhtml

Supplementary Material 1

PRISMA-P (Preferred Reporting Items for Systematic review and Meta-Analysis Protocols)

2015 checklist: recommended items to address in a systematic review protocol*

Section and topic	Item No	Checklist item	Page number
**ADMINISTRATIVE INFORMATION**			
Title:			
Identification	1a	Identify the report as a protocol of a systematic review	1
Update	1b	If the protocol is for an update of a previous systematic review, identify as such	NA
Registration	2	If registered, provide the name of the registry (such as PROSPERO) and registration number	3
Authors:			
Contact	3a	Provide name, institutional affiliation, e-mail address of all protocol authors; provide physical mailing address of corresponding author	1-2
Contributions	3b	Describe contributions of protocol authors and identify the guarantor of the review	18
Amendments	4	If the protocol represents an amendment of a previously completed or published protocol, identify as such and list changes; otherwise, state plan for documenting important protocol amendments	NA
Support:			
Sources	5a	Indicate sources of financial or other support for the review	18
Sponsor	5b	Provide name for the review funder and/or sponsor	18
Role of sponsor or funder	5c	Describe roles of funder(s), sponsor(s), and/or institution(s), if any, in developing the protocol	18
**INTRODUCTION**			
Rationale	6	Describe the rationale for the review in the context of what is already known	6
Objectives	7	Provide an explicit statement of the question(s) the review will address with reference to participants, interventions, comparators, and outcomes (PICO)	6-7
**METHODS**			
Eligibility criteria	8	Specify the study characteristics (such as PICO, study design, setting, time frame) and report characteristics (such as years considered, language, publication status) to be used as criteria for eligibility for the review	7-8

For peer review only

Information sources	9	Describe all intended information sources (such as electronic databases, contact with study authors, trial registers or other grey literature sources) with planned dates of coverage	8-9
Search strategy	10	Present draft of search strategy to be used for at least one electronic database, including planned limits, such that it could be repeated	9; Appendix 2
Study records:			
Data management	11a	Describe the mechanism(s) that will be used to manage records and data throughout the review	9-11
Selection process	11b	State the process that will be used for selecting studies (such as two independent reviewers) through each phase of the review (that is, screening, eligibility and inclusion in meta-analysis)	9-11
Data collection process	11c	Describe planned method of extracting data from reports (such as piloting forms, done independently, in duplicate), any processes for obtaining and confirming data from investigators	10-11
Data items	12	List and define all variables for which data will be sought (such as PICO items, funding sources), any pre-planned data assumptions and simplifications	10-11
Outcomes and prioritization	13	List and define all outcomes for which data will be sought, including prioritization of main and additional outcomes, with rationale	10-11
Risk of bias in individual studies	14	Describe anticipated methods for assessing risk of bias of individual studies, including whether this will be done at the outcome or study level, or both; state how this information will be used in data synthesis	11-12
Data synthesis	15a	Describe criteria under which study data will be quantitatively synthesised	12-13
	15b	If data are appropriate for quantitative synthesis, describe planned summary measures, methods of handling data and methods of combining data from studies, including any planned exploration of consistency (such as I^2, Kendall's τ)	13
	15c	Describe any proposed additional analyses (such as sensitivity or subgroup analyses, meta-regression)	13
	15d	If quantitative synthesis is not appropriate, describe the type of summary planned	13
Meta-bias(es)	16	Specify any planned assessment of meta-bias(es) (such as publication bias across studies, selective reporting within studies)	11-12
Confidence in cumulative evidence	17	Describe how the strength of the body of evidence will be assessed (such as GRADE)	11-12

* It is strongly recommended that this checklist be read in conjunction with the PRISMA-P Explanation and Elaboration (cite when available) for important clarification on the items. Amendments to a review protocol should be tracked and dated. The copyright for PRISMA-P

For peer review only - http://bmjopen.bmj.com/site/about/guidelines.xhtml

(including checklist) is held by the PRISMA-P Group and is distributed under a Creative Commons Attribution Licence 4.0. *From: Shamseer L, Moher D, Clarke M, Ghersi D, Liberati A, Petticrew M, Shekelle P, Stewart L, PRISMA-P Group. Preferred reporting items for systematic review and meta-analysis protocols (PRISMA-P) 2015: elaboration and explanation. BMJ. 2015 Jan 2;349(jan02 1):g7647.*

For peer review only

For peer review only

For peer review only - http://bmjopen.bmj.com/site/about/guidelines.xhtml

Supplementary Material 2 - Electronic search strategy

Ovid MEDLINE, Embase, PsycINFO

1. (ecological adj1 momentary adj1 assessment*).ti,ab.
2. (intensive adj1 longitudinal).ti,ab.
3. (ambulatory adj1 assessment*).ti,ab.
4. (experience adj1 sampl*).ti,ab.
5. (daily adj1 diar*).ti,ab.
6. (ecological adj1 momentary adj1 intervention).ti,ab.
7. within-person.ti,ab.
8. within-subject*.ti,ab.
9. (single adj1 case).ti,ab.
10. idiographic.ti,ab.
11. intraindividual.ti,ab.
12. 1 OR 2 OR 3 OR 4 OR 5 OR 6 OR 7 OR 8 OR 9 OR 10 OR 11
13. tobacco.ti,ab.
14. smok*.ti,ab.
15. cigarette.ti,ab.
16. alcohol*.ti,ab.
17. drinking.ti,ab.
18. addict*.ti,ab.
19. diet.ti,ab.
20. weight.ti,ab.
21. overweight.ti,ab.
22. obes*.ti,ab.
23. (healthy adj3 eat*).ti,ab.
24. physical activity.ti,ab.
25. exercise.ti,ab.
26. sedentary.ti,ab.
27. sitting.ti,ab.
28. leisure.ti,ab.
29. (sexual adj1 health).ti,ab.
30. condom.ti,ab.
31. contraceptive.ti,ab.
32. 13 OR 14 OR 15 OR 16 OR 17 OR 18 OR 19 OR 20 OR 21 OR 22 OR 23 OR 24 OR 25 OR 26 OR 27 OR 28 OR 29 OR 30 OR 31
33. 12 AND 32

Results: 18,014

Web of Science

1. TS=(ecological NEAR/1 momentary NEAR/1 assessment*)
2. TS=(ecological NEAR/1 momentary NEAR/1 intervention)
3. TS=(intensive NEAR/1 longitudinal)
4. TS=(ambulatory NEAR/1 assessment*)
5. TS=(experience NEAR/1 sampl*)
6. TS=(daily NEAR/1 diar*)
7. TS=(within-person or within-subject* or idiographic or intraindividual)

For peer review only

8. TS=(single NEAR/1 case)
9. 1 OR 2 OR 3 OR 4 OR 5 OR 6 OR 7 OR 8
10. TS=(healthy NEAR/1 eat*)
11. TS=(sexual NEAR/1 health)
12. TS=(smok* or tobacco* or cigarette or alcohol* or drinking or addict* or diet or weight or overweight or obes* or physical activity or exercise or sedentary or leisure or sitting or condom or contraceptive)
13. 10 OR 11 OR 12
14. 9 AND 13

Results: 11,036

For peer review only

Systematic review of Ecological Momentary Assessment (EMA) studies of five public health-related behaviours: Review Protocol.

Journal:	*BMJ Open*
Manuscript ID	bmjopen-2020-046435.R2
Article Type:	Protocol
Date Submitted by the Author:	09-Jun-2021
Complete List of Authors:	Kwasnicka, Dominika; SWPS University of Social Sciences and Humanities Wroclaw Campus Library, Faculty of Psychology<br>Kale, Dimitra; University College London<br>Schneider, Verena; University College London<br>Keller, Jan; Freie Universität Berlin<br>Yeboah-Asiamah Asare, Bernard; Curtin University<br>Powell, Daniel; University of Aberdeen<br>Naughton, Felix; University of East Anglia Faculty of Medicine and Health Sciences, School of Health Sciences; University of East Anglia<br>ten Hoor, Gill A. ; Department of Work & Social Psychology, Maastricht University,<br>Verboon, Peter; Open Universiteit Nederland Faculteit Managementwetenschappen<br>Perski, Olga; University College London, Department of Behavioural Science and Health
**Primary Subject Heading**:	Public health
Secondary Subject Heading:	Public health
Keywords:	PUBLIC HEALTH, STATISTICS & RESEARCH METHODS, SOCIAL MEDICINE, SPORTS MEDICINE, NUTRITION & DIETETICS

SCHOLARONE™
Manuscripts

I, the Submitting Author has the right to grant and does grant on behalf of all authors of the Work (as defined in the below author licence), an exclusive licence and/or a non-exclusive licence for contributions from authors who are: i) UK Crown employees; ii) where BMJ has agreed a CC-BY licence shall apply, and/or iii) in accordance with the terms applicable for US Federal Government officers or employees acting as part of their official duties; on a worldwide, perpetual, irrevocable, royalty-free basis to BMJ Publishing Group Ltd ("BMJ") its licensees and where the relevant Journal is co-owned by BMJ to the co-owners of the Journal, to publish the Work in this journal and any other BMJ products and to exploit all rights, as set out in our licence.

The Submitting Author accepts and understands that any supply made under these terms is made by BMJ to the Submitting Author unless you are acting as an employee on behalf of your employer or a postgraduate student of an affiliated institution which is paying any applicable article publishing charge ("APC") for Open Access articles. Where the Submitting Author wishes to make the Work available on an Open Access basis (and intends to pay the relevant APC), the terms of reuse of such Open Access shall be governed by a Creative Commons licence – details of these licences and which Creative Commons licence will apply to this Work are set out in our licence referred to above.

Other than as permitted in any relevant BMJ Author's Self Archiving Policies, I confirm this Work has not been accepted for publication elsewhere, is not being considered for publication elsewhere and does not duplicate material already published. I confirm all authors consent to publication of this Work and authorise the granting of this licence.

For peer review only

Title: Systematic review of Ecological Momentary Assessment (EMA) studies of five public health-related behaviours: Review Protocol.

Authors: Dominika Kwasnicka[1,2], Dimitra Kale[3], Verena Schneider[3], Jan Keller[4], Bernard Yeboah-Asiamah Asare[5,6], Daniel Powell[6,7], Felix Naughton[8], Gill ten Hoor[9], Peter Verboon[10], Olga Perski[3]

1. Faculty of Psychology, SWPS University of Social Sciences and Humanities, Aleksandra Ostrowskiego 30b, 53-238, Wroclaw, Poland

2. NHMRC CRE in Digital Technology to Transform Chronic Disease Outcomes, Melbourne School of Population and Global Health, University of Melbourne, 333 Exhibition Street, 3000, Melbourne, Australia

3. Department of Behavioural Science and Health, Institute of Epidemiology and Health, Faculty of Population Health Sciences, University College London, United Kingdom

4. Department of Education and Psychology, Freie Universität Berlin, Berlin, Germany

5. School of Public Health, Curtin University, Kent Street, 6102, Perth, Australia

6. Health Psychology, Institute of Applied Health Sciences, University of Aberdeen, Aberdeen, AB25 2ZD, United Kingdom

7. Rowett Institute, University of Aberdeen, Aberdeen, AB25 2ZD, United Kingdom

8. Behavioural and Implementation Science Research Group, School of Health Sciences, University of East Anglia, Norwich, NR4 7UL, United Kingdom

9. Department of Work and Social Psychology, Faculty of Psychology and Neurosciences, Maastricht University, Maastricht, The Netherlands

For peer review only - http://bmjopen.bmj.com/site/about/guidelines.xhtml

10. Faculty of Psychology, Open University, Heerlen, The Netherlands

Corresponding author: Dominika Kwasnicka, Faculty of psychology SWPS University of Social Sciences and Humanities, Aleksandra Ostrowskiego 30b, 53-238, Wrocław and NHMRC CRE in Digital Technology to Transform Chronic Disease Outcomes, Melbourne School of Population and Global Health, University of Melbourne, 333 Exhibition Street, 3000, Melbourne, Australia. E-mail: dkwasnicka@swps.edu.pl

Word count: 3,446

Keywords: ambulatory assessment, Ecological Momentary Assessment, EMA, experience sampling, health, psychology, systematic review, within-person design

For peer review only

ABSTRACT

Introduction: Ecological Momentary Assessment (EMA) involves repeated, real-time assessments of phenomena (e.g., cognitions, emotions, behaviours) over a period of time in naturalistic settings. EMA is increasingly used to study both within- and between-person processes. We will review EMA studies investigating key health behaviours and synthesise: 1) study characteristics (e.g., frequency of assessments, adherence, incentives), 2) associations between psychological predictors and behaviours, and 3) moderators of adherence to EMA protocols.

Methods and analysis: This review will focus on EMA studies conducted across five public health behaviours in adult, non-clinical populations: movement behaviour (including physical activity and sedentary behaviour), dietary behaviour, alcohol consumption, tobacco smoking, and preventive sexual health behaviours. Studies need to have assessed at least one psychological or contextual predictor of these behaviours. Studies reporting exclusively on physiological outcomes (e.g., cortisol) or those not conducted under free-living conditions will be excluded. We will search OVID Medline, Embase, PsycINFO and Web of Science using terms relevant to EMA and the selected health behaviours. Reference lists of existing systematic reviews of EMA studies will be hand searched. Identified articles will be screened by two reviewers. This review is expected to provide a comprehensive summary of EMA studies assessing psychological or contextual predictors of five public health behaviours.

Ethics and dissemination: The results will be disseminated through peer-reviewed publications and presentations. Data from included studies will be made available to other researchers. No ethics are required.

Review registration: The review protocol has been registered with PROSPERO 2020 CRD42020168314. Available from: www.crd.york.ac.uk/prospero/display_record.php?ID=CRD42020168314.

For peer review only - http://bmjopen.bmj.com/site/about/guidelines.xhtml

Strengths and limitations of this study:

- A protocol for systematic review is provided for EMA studies in adult, non-clinical populations.
- We included EMA studies of five key public health behaviours including movement behaviours, dietary behaviours, alcohol consumption, tobacco smoking, and preventive sexual health behaviours.
- We will review characteristics of EMA studies (e.g., study duration in days, incentives, adherence rates) and associations between psychological predictors and behaviours, examining rates of adherence to EMA protocols across different health behaviours and moderators of adherence (e.g., study setting, type of behaviour).
- Extracted data will be made available to other researchers, thus allowing for the exploration of additional research questions and potential for setting up a 'living review'.
- As included studies are likely to be heterogeneous, this may limit the overarching conclusions that can be drawn, and will likely prevent meta-analysis combining effect sizes from multiple studies and across all behaviours.

For peer review only

INTRODUCTION

Ecological Momentary Assessment (EMA), also known as ambulatory assessment or experience sampling methodology, involves repeated, real-time assessments of phenomena (e.g., cognitions, emotions and behaviours) over a period of time in naturalistic settings (1). EMA is increasingly used to study within- and between-person processes, including associations between psychological and health behaviour-related variables (e.g., positive affect and physical activity in general population or stress and lapse risk in smokers attempting to stop). For researchers and healthcare professionals to understand and change behaviour, it is important for theories and interventions to be applicable to both momentary states of individuals (within-person processes) and groups of individuals (between-person processes) (2). Despite the popularity and importance of EMA for studying health-related behaviours, there has been no comprehensive systematic investigation of characteristics of EMA studies (e.g., rates of adherence, study duration in days, incentive schedules) and potential moderators of adherence (e.g., study setting, type of health behaviour), with attempts to describe associations between psychological predictors (e.g., intentions, self-efficacy) and key public health behaviours.

Previous reviews of EMA studies have focused on clinical conditions such as borderline personality disorder (3), psychotic disorder (4), mood disorders (5), binge eating (6), bulimia nervosa (7), anxiety disorder (8), schizophrenia (9), alcohol use disorder (10), chronic pain (11), and specific populations such as children and adolescents (12), youth (13) and older adults (14). Health behaviour-specific reviews of EMA studies have focused on physical activity (15,16), sedentary behaviour (16), alcohol use (17), craving and substance use (18), dietary behaviours (19), and the relationship between alcohol use and sexual decision making (20). Previous EMA reviews have also focused on interrelations between specific psychological

For peer review only - http://bmjopen.bmj.com/site/about/guidelines.xhtml

variables, such as the association of everyday social interactions with intra-individual variability in affect (21).

While systematic reviews of EMA studies focusing on specific health behaviours have been conducted (18,22,23), there are no overarching reviews that can help address broad questions about study characteristics (e.g., study duration in days, adherence, incentive schedules), rates and moderators of adherence (e.g., study setting, participant characteristics) and predictor-behaviour associations across different health behaviours and potential moderators (e.g., study setting, study quality). It is expected that this review will help fill this gap. We also expect that this review will help inform the design of future EMA studies by providing a summary of best practice across research contexts, settings and health-related behaviours. For instance, results may be useful for informing researchers' understanding of what frequency or intensity of change we would expect to see at what temporal resolution [i.e., informed by a 'theory of change' (24)], which can then inform assessment scheduling decisions. This review is likely to include a large number of studies, thus providing a comprehensive overview of the EMA literature.

The current study

We will synthesise evidence from EMA studies that report either within- or between-person predictor-behaviour associations. The review will focus on five key public health behaviours: (1) movement behaviours (including physical activity and sedentary behaviour), (2) dietary behaviours, (3) alcohol consumption, (4) tobacco smoking and (5) preventive sexual health behaviours (including contraceptive use).

The review aims are:

1. To summarise adherence to EMAs, total length of data collection of EMAs, prompting frequency of EMAs, and incentives structures across studies.

For peer review only

2. To describe within- and between-person predictor-behaviour associations across EMA studies (e.g., associations between intention and behaviour).
3. To assess potential moderators of adherence to EMAs (e.g., study setting, participant characteristics).

This review is intentionally broad in scope to provide an overview of the field for researchers interested in the application of EMAs to the study of health-related behaviours. We expect this overarching review to help identify patterns and key knowledge gaps.

For peer review only

METHODS AND ANALYSIS

Study design

This review will adhere to the Preferred Reporting Items for Systematic Reviews and Meta-Analyses (PRISMA) checklist (Supplementary Material 1). The review start date was 15th September 2019 and the planned end date is 30th December 2021.

Inclusion criteria

This review will focus on five key public health behaviours in healthy adults (i.e. non-clinical populations) aged 18+ years, namely:

1. movement behaviours, including physical activity and sedentary behaviour;
2. dietary behaviours, including snacking or fruit and vegetable consumption;
3. alcohol consumption;
4. tobacco smoking, including cigarette, cigar or pipe smoking;
5. preventive sexual health behaviours, including contraceptive/condom use.

No restrictions on geographical location or publication date will be set. To be included, studies need to incorporate multiple (i.e., two or more) within-day, daily or weekly assessments of predictors and behaviours, and to have reported either (or both) within- or between-person predictor-behaviour (e.g., stress predicting unhealthy snack consumption) associations. The

For peer review only - http://bmjopen.bmj.com/site/about/guidelines.xhtml

frequency of the EMAs should plausibly match how the target behaviour (and psychological and contextual predictors) theoretically or empirically unfolds over time, e.g., daily assessments of steps, weekly assessments of gym class attendance if the class is undertaken only once a week. To be included, studies need to assess one of the aforementioned behaviours and at least one psychological or contextual variable via EMAs.

In this review, we defined psychological variables as emergent properties of a distributed network of neurons, including cognition (e.g., beliefs, attitudes, goals), emotion (e.g., negative affect, cravings) and processes operating on these (e.g., self-regulation, learning), which are linked to behaviour. We further define contextual variables as any potential environmental (e.g., social or physical) influences on behaviour, including the presence of other people, weather, or the availability of unhealthy foods/cigarettes/alcohol. The psychological and contextual variables will be closely assessed by the reviewers as to their suitability for inclusion/exclusion in the review.

In addition to self-report measures, included studies can use physiological measures of psychological predictors (e.g., cortisol or heart rate variability to measure stress) or behaviours (e.g., accelerometer data to measure physical activity or sedentary behaviour). Studies reporting associations between behaviours and psychological consequences (e.g., whether physical activity predicts affect) will be included providing that they also report psychological or contextual predictor-behaviour associations (e.g., whether positive affect predicts physical activity). We will include individuals with overweight and obesity given that 39% of adults globally fall into this category, with most Western countries averaging above 50% (25). Studies including participants with a diagnosed mental or physical health condition who were not recruited into the study on the basis of their condition will be included (e.g., studies including participants with clinical levels of depression but where this was not an inclusion criterion).

For peer review only - http://bmjopen.bmj.com/site/about/guidelines.xhtml

Studies in which a behavioural or pharmacological intervention was delivered will be included providing that participants were asked to complete free-living EMAs.

Exclusion criteria

Laboratory studies will not be included. Studies examining clinical populations, that is, solely recruiting participants on the basis of being diagnosed with a physical or mental health condition such as cancer, cardiovascular disease, depression, binge eating disorder or substance use disorder (also including case-control studies) will be excluded. Studies focusing only on purchasing behaviours (e.g., tobacco purchasing, food purchasing) will not be included. Studies focusing on e-cigarettes will be also excluded. Studies not published in English or where no full text could be obtained will also not be included. Although behaviour-behaviour associations may also be considered relevant, our electronic search is not designed to capture such studies, and behaviour-behaviour associations will hence not be considered further in this review.

Search methods for the identification of studies

Electronic searches

We will search Ovid MEDLINE, Embase, PsycINFO and Web of Science (see Supplementary Material 2 for the full search strategy). Terms will be searched in titles and abstracts as free text terms or as index terms (e.g., Medical Subject Headings), as appropriate. We will combine two groups of terms, the first with terms relevant to EMAs and within-person study designs; the second with terms relevant to the five health behaviours addressed in this review.

Example terms used:

1. (ecological adj1 momentary adj1 assessment*) OR (intensive adj1 longitudinal) OR (ambulatory adj1 assessment*) OR (experience adj1 sampl*) OR (daily adj1 diar*) OR (ecological adj1 momentary adj1 intervention) OR within-person OR within-subject* OR (single adj1 case) OR idiographic OR intraindividual

For peer review only - http://bmjopen.bmj.com/site/about/guidelines.xhtml

2. tobacco OR smok* OR cigarette OR alcohol* OR drinking OR addict* OR (healthy adj3 eat*) OR diet OR weight OR overweight OR obes* OR physical activity OR exercise OR sedentary OR sitting OR leisure OR (sexual adj1 health) OR condom OR contraceptive
3. 1 AND 2

Electronic and hand searches were conducted in January 2020 and updated in February 2021. We restricted the search to human studies available in English that are published in peer-reviewed journals (Supplementary Material 2).

Searching for other sources

Reference lists of existing systematic reviews of EMA studies will be hand searched and expertise within the review team will be used to identify additional articles of interest.

Data collection and analysis

Selection of studies

Identified articles will be merged using Covidence (26) and duplicate records will be removed. The three lead authors (DK, OP and JK) will independently screen titles and abstracts (yes, maybe, no) against the pre-specified inclusion criteria. Full texts will be screened by two reviewers independently (yes, no); discrepancies will be resolved by the lead authors and inclusion will be further discussed with other team members if needed. In line with the PRISMA checklist, key reasons for exclusion will be recorded at the full text stage. These will include: lack of psychological predictors or outcomes; study not being relevant to the five key public health behaviours of interest; wrong study design (not an EMA study); participants being recruited based on a health condition (i.e., clinical population); participants younger than 18 years old; studies of purchasing behaviours; conference abstracts; protocols; duplicates; studies not published in English or no full text could be obtained. We will follow the hierarchy

For peer review only - http://bmjopen.bmj.com/site/about/guidelines.xhtml

of the exclusion criteria, listing the first reason from the aforementioned list as the key reason for exclusion.

Data extraction and management

A data extraction form will be developed in Microsoft Excel to extract information and to import data into R for analysis. Each study will be allocated a unique study identification number. Data will be extracted on:

- *Study description* (study author, year, country, study funder);
- *Participant characteristics* (sample size; mean or median age (SD); gender (% female); educational attainment (% university education); population type (e.g., men who have sex with men, older adults, general population), ethnicity (% White ethnicity);
- *EMA study type* (e.g., observational, interventional, both);
- *EMA delivery mode* (e.g., mobile phone, website/online, pen-and-paper);
- *EMA method* (e.g., signal contingent, event contingent, multiple);
- *EMA characteristics* (e.g., total study duration in days; prompting frequency (e.g., hourly, daily, weekly), incentive schedule (e.g., flat rate, payment per EMA);
- *Adherence to EMA* (e.g., average % EMAs completed out of available prompts);
- *Health behaviour(s) assessed* (e.g., physical activity, sedentary behaviour, dietary behaviour, tobacco smoking); and how the health behaviour(s) were measured (e.g., hourly step count, number of cigarettes smoked per day);
- *Psychological and contextual predictors* (e.g., intentions, self-efficacy, presence of other smokers) and how they were measured (e.g., EMA method, measurement frequency, whether the measure was developed for the study (versus precedent), whether a single item or multiple items were used);

For peer review only - http://bmjopen.bmj.com/site/about/guidelines.xhtml

- *Statistical model used to examine predictor-behaviour association* (e.g. multilevel model, generalised estimating equation) and whether these associations were analysed on the within- and/or between-level;
- *Level of aggregation in data analysis* (i.e. whether data underpinning the predictor-behaviour association are aggregated vs. maintained at the within-person level);
- *Coefficients and effect sizes from statistical models* (e.g. odds ratios, relative risks, regression coefficients);
- *Control variables in multivariate models* (e.g. age, sex)

For each study, one reviewer will extract the data. At least 20% of studies stratified by behaviour (e.g., 20% of all alcohol consumption studies) will be double checked for accuracy and completeness by a second reviewer. In case there are any uncertainties related to data extraction (e.g., the primary data extractor is uncertain about a particular parameter or a large number of discrepancies are observed across the primary and secondary data extractor), we will double check additional studies until agreement is achieved. All review authors will be involved in data extraction and double checking.

Quality appraisal

Included studies may vary in quality, which will be considered through a quality appraisal. The appraisal tool was developed by the review team, based on an existing EMA reporting checklist (27), and includes the following four criteria: 1) rationale for EMA design, 2) a priori power analysis to determine sample size, 3) percentage adherence to the EMA protocol, and 4) treatment of missingness (Table 1). The quality indicators will be coded by one reviewer, with 20% or more double checked by a second reviewer. Discrepancies will be resolved through discussion among the lead authors. Where possible, each study quality indicator will be entered as a moderator of predictor-behaviour associations. As each criterion

For peer review only - http://bmjopen.bmj.com/site/about/guidelines.xhtml

refers to a different aspect of study quality, we will not summarise study quality, but will present how studies score on each selected dimension.

Topic: Factors	Strong	Moderate	Weak
**Rationale**			
***1. Rationale for EMA design provided**:* Why was an EMA design chosen to examine the research question?	A strong rationale provided for the EMA design of predictor AND behaviour/ outcome	Rationale provided but not very strong for the EMA design of either the predictor OR behaviour/ outcome	No rationale for the EMA design regarding predictor and behaviour/ outcome
**Power analysis, sample size and participant adherence**			
***2. Power analysis**:* A priori power analysis to determine sample size	An a priori power analysis is reported and the enrolled sample size met power analysis indication / OR: sufficient explanation as to why an a priori power analysis was not needed	An a priori power analysis is reported but sufficient sample size/number of observations was not achieved	No information about power analysis / OR: a post-hoc power analysis is reported
3. Adherence to EMA protocol: Percentage of answered EMA prompts across all participants for the main EMA study period	Percentage of answered EMA prompts >80%	Percentage of answered EMA prompts 60-79.99%	Percentage of answered EMA prompts less than 60%
**Data analysis**			
4. Treatment of missingness: Report whether study dropout or non-adherence to EMAs (e.g., missed prompts) are related to specific variables	Missing mechanisms/predictors are identified, reported and mitigated for if needed	Missing mechanisms/predictors are identified and reported but not mitigated for	Missing mechanisms/predictors are not identified or reported

Table 1. Quality appraisal in included EMA studies.

Data synthesis

All quantitative analyses will be conducted in R v.3.5.1. A narrative (descriptive) synthesis will be conducted. We will summarise the number of EMA studies conducted for

For peer review only - http://bmjopen.bmj.com/site/about/guidelines.xhtml

each of the five health behaviours, study setting (e.g., country, immediate study setting), and sample size (i.e. mean or median number of participants per study). We will then present results in relation to each research question.

To address the first aim, we will summarise study and EMA characteristics, e.g., study setting, population characteristics, percentage prompting frequency (e.g., % daily, % weekly), percentage type of EMA method (e.g., % event contingent, % random assessments, % continuous sensor based, % hybrid), percentage type of EMA delivery mode (e.g., % smartphone app delivery), percentage type of incentive structure (e.g., % flat payment, % payment per EMA, % no incentive), rates of EMA adherence (mean or median), and study duration (mean or median).

To address the second aim, we will summarise within- and between-person predictor-behaviour associations across EMA studies (e.g., the type of psychological or contextual predictor assessed, measurement type, frequency of measurement). If there is sufficient homogeneity between studies (e.g., similar predictors assessed with similar measurement type and frequency across ≥3 studies), within- or between-person predictor-behaviour associations (e.g., odds ratios, relative risks, regression coefficients) will be synthesised with random effects meta-analyses, grouped by behaviour. Analyses will be conducted with the 'metafor' or ‘CTmeta’ packages (28-30), as appropriate, also utilising ‘jamovi’ (31). Where sufficient detail on model parameter estimates is lacking in the publications, we may contact study authors to request access to additional information.

To address the third aim, we will assess, with regression analyses, whether EMA adherence varies depending on study setting, study characteristics, participant characteristics, or type of incentive schedule used. We do not have any pre-specified hypotheses. Where appropriate, moderator analyses will be conducted to examine whether predictor-behaviour

associations vary depending on study setting, study characteristics, participant characteristics, or type of incentive schedule used.

Patient and public involvement

A patient and public involvement representative reviewed a lay summary of the protocol for our systematic review. Positive feedback was received on the review's aims, the importance of the current research and choice of key behaviours relevant to public health. Once the review is completed, feedback will be sought from the additional patient and public involvement representatives about the interpretation of findings and plans for dissemination. We will seek advice on how to best present the study outcomes and use them in order to design studies and interventions that are useful and relevant for the public.

ETHICS AND DISSEMINATION

This study does not require ethics approval as it will summarise data from previously published studies. A protocol was pre-registered on the international Prospective Register of Systematic Reviews (PROSPERO) and on the Open Science Framework; it will also be offered for peer-review and publication in an open access journal. The findings of the review will be disseminated through peer-reviewed publications and presentations at relevant conferences. The dataset will be made available to other researchers online via the creation of a digital object identifier, thus enabling further research questions to be addressed. We expect this review to be useful for researchers and healthcare practitioners who regularly design and interpret results from EMA studies. We plan to publish overarching review and subsequently five behaviour-specific reviews that will provide a more in-depth synthesis of predictor-behaviour associations.

Summary

EMA is a frequently used research method; however, an overview of studies using this method across key public health behaviours in healthy adults is lacking. This review will

For peer review only

provide a comprehensive overview of associations between a psychological/contextual predictor and a health behaviour in EMA studies focusing on movement behaviours, dietary behaviours, alcohol consumption, tobacco smoking and sexual health behaviours. This review will inform the future design of EMA studies and it will influence practice of assessing individuals in real life settings and providing interventions that are delivered at the time and place when and where required. This review will set a blueprint for how to conduct EMA studies to improve participants' adherence and conduct meaningful studies in real life settings.

For peer review only

REFERENCES

1. Stone AA, Shiffman S. Ecological momentary assessment (EMA) in behavorial medicine. Ann Behav Med. 1994;

2. Johnston DW, Johnston M. Useful theories should apply to individuals. Br J Health Psychol. 2013;18(3):469–73.

3. Santangelo P, Bohus M, Ebner-Priemer UW. Ecological momentary assessment in borderline personality disorder: a review of recent findings and methodological challenges. J Personal Disord. 2014;28(4):555–76.

4. Bell IH, Lim MH, Rossell SL, Thomas N. Ecological momentary assessment and intervention in the treatment of psychotic disorders: a systematic review. Psychiatr Serv. 2017;68(11):1172–81.

5. aan het Rot M, Hogenelst K, Schoevers RA. Mood disorders in everyday life: A systematic review of experience sampling and ecological momentary assessment studies. Clin Psychol Rev. 2012;32(6):510–23.

6. Haedt-Matt AA, Keel PK. Revisiting the affect regulation model of binge eating: a meta-analysis of studies using ecological momentary assessment. Psychol Bull. 2011;137(4):660.

7. Goldschmidt AB, Wonderlich SA, Crosby RD, Engel SG, Lavender JM, Peterson CB, et al. Ecological momentary assessment of stressful events and negative affect in bulimia nervosa. J Consult Clin Psychol. 2014;82(1):30.

8. Walz LC, Nauta MH, aan het Rot M. Experience sampling and ecological momentary assessment for studying the daily lives of patients with anxiety disorders: A systematic review. J Anxiety Disord. 2014;28(8):925–37.

9. Mote J, Fulford D. Ecological momentary assessment of everyday social experiences of people with schizophrenia: A systematic review. Schizophr Res. 2020;216:56–68.

10. Morgenstern J, Kuerbis A, Muench F. Ecological momentary assessment and alcohol use disorder treatment. Alcohol Res Curr Rev. 2014;36(1):101.

11. May M, Junghaenel DU, Ono M, Stone AA, Schneider S. Ecological momentary assessment methodology in chronic pain research: a systematic review. J Pain. 2018;19(7):699–716.

12. Wen CKF, Schneider S, Stone AA, Spruijt-Metz D. Compliance with mobile ecological momentary assessment protocols in children and adolescents: a systematic review and meta-analysis. J Med Internet Res. 2017;19(4):e132.

13. Heron KE, Everhart RS, McHale SM, Smyth JM. Using mobile-technology-based ecological momentary assessment (EMA) methods with youth: A systematic review and recommendations. J Pediatr Psychol. 2017;42(10):1087–107.

14. Cain AE, Depp CA, Jeste DV. Ecological momentary assessment in aging research: a critical review. J Psychiatr Res. 2009;43(11):987–96.

For peer review only - http://bmjopen.bmj.com/site/about/guidelines.xhtml

15. Dunton GF. Ecological momentary assessment in physical activity research. Exerc Sport Sci Rev. 2017;45(1):48.

16. Degroote L, DeSmet A, De Bourdeaudhuij I, Van Dyck D, Crombez G. Content validity and methodological considerations in ecological momentary assessment studies on physical activity and sedentary behaviour: a systematic review. Int J Behav Nutr Phys Act. 2020;17(1):1–13.

17. Wray TB, Merrill JE, Monti PM. Using ecological momentary assessment (EMA) to assess situation-level predictors of alcohol use and alcohol-related consequences. Alcohol Res Curr Rev. 2014;36(1):19.

18. Serre F, Fatseas M, Swendsen J, Auriacombe M. Ecological momentary assessment in the investigation of craving and substance use in daily life: a systematic review. Drug Alcohol Depend. 2015;148:1–20.

19. Maugeri A, Barchitta M. A systematic review of ecological momentary assessment of diet: implications and perspectives for nutritional epidemiology. Nutrients. 2019;11(11):2696.

20. Scott-Sheldon LA, Carey KB, Cunningham K, Johnson BT, Carey MP, MASH Research Team. Alcohol use predicts sexual decision-making: a systematic review and meta-analysis of the experimental literature. AIDS Behav. 2016;20(1):19–39.

21. Liu H, Xie QW, Lou VW. Everyday social interactions and intra-individual variability in affect: A systematic review and meta-analysis of ecological momentary assessment studies. Motiv Emot. 2019;43(2):339–53.

22. Schembre SM, Liao Y, O'connor SG, Hingle MD, Shen S-E, Hamoy KG, et al. Mobile ecological momentary diet assessment methods for behavioral research: Systematic review. JMIR MHealth UHealth. 2018;6(11):e11170.

23. Jones A, Remmerswaal D, Verveer I, Robinson E, Franken IHA, Wen CKF, et al. Compliance with ecological momentary assessment protocols in substance users: a meta-analysis. Addiction. 2019 Apr;114(4):609–19.

24. Collins LM. Analysis of longitudinal data: The integration of theoretical model, temporal design, and statistical model. Annu Rev Psychol. 2006;57:505–28.

25. WHO. Obesity and overweight [Internet]. 2018 [cited 2020 Jan 11]. Available from: https://www.who.int/news-room/fact-sheets/detail/obesity-and-overweight

26. Veritas Health Innovation. Covidence systematic review software. 2016;

27. Liao Y, Skelton K, Dunton G, Bruening M. A Systematic Review of Methods and Procedures Used in Ecological Momentary Assessments of Diet and Physical Activity Research in Youth: An Adapted STROBE Checklist for Reporting EMA Studies (CREMAS). J Med Internet Res. 2016 Jun 21;18(6):e151.

28. Balaji Y, Sankaranarayanan S, Chellappa R. Metareg: Towards domain generalization using meta-regularization. In 2018. p. 998–1008.

For peer review only

29. Viechtbauer W. Metafor: meta-analysis package for R. R Package Version. 2010;2010:1–0.

30. Kuiper RM, Ryan O. Meta-analysis of Lagged Regression Models: A Continuous-time Approach. Struct Equ Model Multidiscip J. 2020 May 3;27(3):396–413.

31. Sahin MD, Aybek EC. Jamovi: An Easy to Use Statistical Software for the Social Scientists. Int J Assess Tools Educ. 2019;6(4):670–92.

For peer review only

Authors' contributions

DK, OP, DP and FN conceived the project. DK and OP are the project leads and coordinators, they jointly drafted the manuscript. All authors (DK, DK, VS, JK, BYAA, DP, FN, GH, PV, OP) have made conceptual contributions to project design and procedures. All authors read, edited and approved the final version.

Funding

Dominika Kwasnicka's work is carried out within the HOMING program of the Foundation for Polish Science co-financed by the European Union under the European Regional Development Fund; grant number POIR.04.04.00-00-5CF3/18-00; HOMING 5/2018. Dimitra Kale and Olga Perski receive salary support from Cancer Research UK (C1417/A22962). Daniel Powell is funded by the Scottish Government's Rural and Environment Science and Analytical Services (RESAS) and by the School of Medicine, Medical Sciences, and Nutrition (SMMSN) at the University of Aberdeen. Felix Naughton's salary is covered by the Faculty of Medicine and Health Sciences at the University of East Anglia.

Acknowledgments

The review team would like to thank Dr Marta Marques for comments and suggestions on the initial version of the review protocol. The authors would like to thank the patient and public involvement representative who commented on the lay summary of our proposed plan, for their contribution to this research.

Competing interests

The authors have no competing interests to declare.

Data statement

Data associated with this manuscript will be available on OSF.

For peer review only

Supplementary Material 1

PRISMA-P (Preferred Reporting Items for Systematic review and Meta-Analysis Protocols)

2015 checklist: recommended items to address in a systematic review protocol*

Section and topic	Item No	Checklist item	Page number
**ADMINISTRATIVE INFORMATION**			
Title:			
Identification	1a	Identify the report as a protocol of a systematic review	1
Update	1b	If the protocol is for an update of a previous systematic review, identify as such	NA
Registration	2	If registered, provide the name of the registry (such as PROSPERO) and registration number	3
Authors:			
Contact	3a	Provide name, institutional affiliation, e-mail address of all protocol authors; provide physical mailing address of corresponding author	1-2
Contributions	3b	Describe contributions of protocol authors and identify the guarantor of the review	18
Amendments	4	If the protocol represents an amendment of a previously completed or published protocol, identify as such and list changes; otherwise, state plan for documenting important protocol amendments	NA
Support:			
Sources	5a	Indicate sources of financial or other support for the review	18
Sponsor	5b	Provide name for the review funder and/or sponsor	18
Role of sponsor or funder	5c	Describe roles of funder(s), sponsor(s), and/or institution(s), if any, in developing the protocol	18
**INTRODUCTION**			
Rationale	6	Describe the rationale for the review in the context of what is already known	6
Objectives	7	Provide an explicit statement of the question(s) the review will address with reference to participants, interventions, comparators, and outcomes (PICO)	6-7
**METHODS**			
Eligibility criteria	8	Specify the study characteristics (such as PICO, study design, setting, time frame) and report characteristics (such as years considered, language, publication status) to be used as criteria for eligibility for the review	7-8

For peer review only

Information sources	9	Describe all intended information sources (such as electronic databases, contact with study authors, trial registers or other grey literature sources) with planned dates of coverage	8-9
Search strategy	10	Present draft of search strategy to be used for at least one electronic database, including planned limits, such that it could be repeated	9; Appendix 2
Study records:			
Data management	11a	Describe the mechanism(s) that will be used to manage records and data throughout the review	9-11
Selection process	11b	State the process that will be used for selecting studies (such as two independent reviewers) through each phase of the review (that is, screening, eligibility and inclusion in meta-analysis)	9-11
Data collection process	11c	Describe planned method of extracting data from reports (such as piloting forms, done independently, in duplicate), any processes for obtaining and confirming data from investigators	10-11
Data items	12	List and define all variables for which data will be sought (such as PICO items, funding sources), any pre-planned data assumptions and simplifications	10-11
Outcomes and prioritization	13	List and define all outcomes for which data will be sought, including prioritization of main and additional outcomes, with rationale	10-11
Risk of bias in individual studies	14	Describe anticipated methods for assessing risk of bias of individual studies, including whether this will be done at the outcome or study level, or both; state how this information will be used in data synthesis	11-12
Data synthesis	15a	Describe criteria under which study data will be quantitatively synthesised	12-13
	15b	If data are appropriate for quantitative synthesis, describe planned summary measures, methods of handling data and methods of combining data from studies, including any planned exploration of consistency (such as I^2, Kendall's τ)	13
	15c	Describe any proposed additional analyses (such as sensitivity or subgroup analyses, meta-regression)	13
	15d	If quantitative synthesis is not appropriate, describe the type of summary planned	13
Meta-bias(es)	16	Specify any planned assessment of meta-bias(es) (such as publication bias across studies, selective reporting within studies)	11-12
Confidence in cumulative evidence	17	Describe how the strength of the body of evidence will be assessed (such as GRADE)	11-12

* It is strongly recommended that this checklist be read in conjunction with the PRISMA-P Explanation and Elaboration (cite when available) for important clarification on the items. Amendments to a review protocol should be tracked and dated. The copyright for PRISMA-P

For peer review only - http://bmjopen.bmj.com/site/about/guidelines.xhtml

(including checklist) is held by the PRISMA-P Group and is distributed under a Creative Commons Attribution Licence 4.0. *From: Shamseer L, Moher D, Clarke M, Ghersi D, Liberati A, Petticrew M, Shekelle P, Stewart L, PRISMA-P Group. Preferred reporting items for systematic review and meta-analysis protocols (PRISMA-P) 2015: elaboration and explanation. BMJ. 2015 Jan 2;349(jan02 1):g7647.*

For peer review only

For peer review only

For peer review only - http://bmjopen.bmj.com/site/about/guidelines.xhtml

Supplementary Material 2 - Electronic search strategy

Ovid MEDLINE, Embase, PsycINFO

1. (ecological adj1 momentary adj1 assessment*).ti,ab.
2. (intensive adj1 longitudinal).ti,ab.
3. (ambulatory adj1 assessment*).ti,ab.
4. (experience adj1 sampl*).ti,ab.
5. (daily adj1 diar*).ti,ab.
6. (ecological adj1 momentary adj1 intervention).ti,ab.
7. within-person.ti,ab.
8. within-subject*.ti,ab.
9. (single adj1 case).ti,ab.
10. idiographic.ti,ab.
11. intraindividual.ti,ab.
12. 1 OR 2 OR 3 OR 4 OR 5 OR 6 OR 7 OR 8 OR 9 OR 10 OR 11
13. tobacco.ti,ab.
14. smok*.ti,ab.
15. cigarette.ti,ab.
16. alcohol*.ti,ab.
17. drinking.ti,ab.
18. addict*.ti,ab.
19. diet.ti,ab.
20. weight.ti,ab.
21. overweight.ti,ab.
22. obes*.ti,ab.
23. (healthy adj3 eat*).ti,ab.
24. physical activity.ti,ab.
25. exercise.ti,ab.
26. sedentary.ti,ab.
27. sitting.ti,ab.
28. leisure.ti,ab.
29. (sexual adj1 health).ti,ab.
30. condom.ti,ab.
31. contraceptive.ti,ab.
32. 13 OR 14 OR 15 OR 16 OR 17 OR 18 OR 19 OR 20 OR 21 OR 22 OR 23 OR 24 OR 25 OR 26 OR 27 OR 28 OR 29 OR 30 OR 31
33. 12 AND 32

Results: 18,014

Web of Science

1. TS=(ecological NEAR/1 momentary NEAR/1 assessment*)
2. TS=(ecological NEAR/1 momentary NEAR/1 intervention)
3. TS=(intensive NEAR/1 longitudinal)
4. TS=(ambulatory NEAR/1 assessment*)
5. TS=(experience NEAR/1 sampl*)
6. TS=(daily NEAR/1 diar*)
7. TS=(within-person or within-subject* or idiographic or intraindividual)

For peer review only

8. TS=(single NEAR/1 case)
9. 1 OR 2 OR 3 OR 4 OR 5 OR 6 OR 7 OR 8
10. TS=(healthy NEAR/1 eat*)
11. TS=(sexual NEAR/1 health)
12. TS=(smok* or tobacco* or cigarette or alcohol* or drinking or addict* or diet or weight or overweight or obes* or physical activity or exercise or sedentary or leisure or sitting or condom or contraceptive)
13. 10 OR 11 OR 12
14. 9 AND 13

Results: 11,036

For peer review only
